# Supplementary material for: Food groups, macronutrient intake and objective measures of total carotenoids and fatty acids in 16-to-24-year-olds following different plant-based diets compared to an omnivorous diet
Source: PLoS One. 2025 Jan 17;20(1):e0311118. doi: 10.1371/journal.pone.0311118 (PMC11741618; doi:10.1371/journal.pone.0311118)
Supplement: S1 Table — (DOCX) [file pone.0311118.s001.docx]

**Supplemental Table 1. Food items included in the food groups in VeggiSkills-Norway.**

| **Food groups** | **Food items included** |
| --- | --- |
| Whole grain products | ‘Oatmeal (uncooked)’, ‘Bread, 75% whole wheat flour, homemade’, ‘Bread, 75% whole wheat flour, water, homemade’, ‘Baguette, whole grain, homemade’, ‘Bread roll, whole grain, with skimmed milk, homemade’, ‘Bread Roll, medium-whole grain, with skimmed milk, homemade’, ‘Bread roll, whole grain, half-baked, purchased, baked’, ‘Bread roll, whole grain, purchased’, ‘Scones, whole grain, with milk’, ‘Bread, gluten-free, whole grain, with water, homemade’, ‘Bread, gluten-free, whole grain, with soy milk, homemade’, ‘Bread, whole grain, 50-75%, purchased’, ‘Bread, extra whole grain, 75-100%, purchased‘, ‘Bread, extra whole grain, 75-100%, purchased (brand: Oeat bread)’, ‘Bread, coarse, 50-75%, purchased ,(brand: Ice Age bread)’, ‘Bread, coarse, 50-75%, purchased, Norwegian Mountain Bread’, ‘Rye bread, whole grain, 50-75%, purchased’, ‘Pumpernickel, whole grain bread, extra coarse, 75-100%, purchased’, ‘Buckwheat, whole grain’, ‘Wheat, whole grain’, ‘Barley, cooked’, ‘Pasta, whole wheat, cooked with salt’, ‘Bulgur, cooked’, ‘Crispbread, oat’, ‘Crispbread, rye, Husman’, ‘Crispbread, homemade’, ‘Crispbread, rye, coarse’, ‘Crispbread, with extra fiber’, ‘Crispbread, rye, thin, Rugsprø, Finncrisp’, ‘Crispbread, rye, with filling, sandwich’, ‘Bread, 1/3 whole wheat flour, water, homemade’, ‘Crispbread, rye, Ryvita’, ‘Oatmeal, with milk’, ‘Oatmeal, with water’, ‘Oat flour’, ‘Rice, wild, cooked’, ‘Rice, unpolished, brown rice, long-grain, cooked’, ‘Quinoa, white, cooked’, ‘Quinoa, cooked’, ‘Quinoa, red, dry’ |
| Refined grain products | ‘Bread, 25% whole wheat flour, water, homemade’, ‘Croissant, fine, homemade’, ‘Roll, medium-coarse, with water, homemade’, ‘Focaccia’, ‘Roll, fine, with skimmed milk, homemade’, ‘Bagels, homemade’, ‘Chapatti, with whole wheat flour’, ‘White bread, fine, 0-25%, baked in a loaf pan, purchased’, ‘Baguette, fine, half-baked, baked’, ‘Tortilla, wraps/burrito, wheat flour’, ‘Hamburger bun, fine, purchased’, ‘Tortilla, corn tortilla’, ‘Potato flatbread, purchased’, ‘Polar bread, wheat’, ‘Tortilla, wraps, wheat flour, Santa Maria’, ‘Pita bread, fine, purchased’, ‘Pasta, plain, fresh, cooked’, ‘Noodles, cooked, with seasoning’, ‘Rice, Basmati, cooked’, ‘Crispbread, light’, ‘Flatbread, homemade type, purchased’, ‘Puff pastry, baked’, ‘Lasagna sheets’, ‘Wheat noodles, egg-free, cooked’, ‘Rice, Jasmine, cooked’, ‘Rice, polished, parboiled, long-grain, cooked’, ‘Glass noodles, cooked’, ‘Rice noodles, cooked without salt’, ‘Flour, gluten-free, light, Toro’, ‘Wonton wrappers’, ‘Glass noodles, cooked’, ‘Couscous, cooked’, ‘Wheat flour, whole, fine/coarse’, ‘Bread, 1/3 whole wheat flour, water, homemade’, ‘Bread, semi-coarse, 25-50%, purchased Kneipp bread’, ‘Bread, semi-coarse, 25-50%, purchased Odels bread’, ‘Wheat flour’, ‘Rice cakes, lightly salted’, ‘Crackers, Captain’, ‘Rice cakes, unsalted’ |
| Vegetables | ‘Onion, yellow/red, raw’, ‘Carrot, raw’, ‘Garlic, raw’, ‘Cucumber, raw’, ‘Cherry tomato, imported, raw’, ‘Lettuce, raw’, ‘Arugula, raw’, ‘Beetroot, raw’, ‘Red pepper, raw’, ’Iceberg lettuce, raw’, ‘Broccoli, raw’, ‘Spinach, raw’, ‘Tomato, imported, raw’, ‘Broccoli, frozen, blanched’, ‘Chinese cabbage, raw’, ‘Shallot, raw’, ‘Chili, red, raw’, ‘Leek, raw’, ‘Zucchini, raw’, ‘Ginger root, raw’, ‘Kale, raw’, ‘Jalapeño, raw’, ‘Cabbage, raw’, ‘Pumpkin, raw’, ‘Artichoke, raw’, ‘Bean sprouts, mung bean sprouts, raw’, ‘Spinach, frozen, raw’, ‘Green onion, raw’, ‘Fennel, raw’, ‘Red cabbage, raw’, ‘Pak-choi, bok choy, cabbage, raw’, ‘Brussels sprouts, frozen, blanched’, ‘Celery stick, raw’, ‘Cauliflower, cooked’, ‘Mushroom, unspecified, fried in fat’, ‘Broccoli, cooked’, ‘Parsnip, cooked’, ‘Carrot, cooked’, ‘Chanterelle, fried in fat’, ‘Red pepper, cooked’, ‘Stir-fry mix, classic, stir-fried without fat’, ‘Stir-fry mix (Indian type, stir-fried without fat)’, ‘Chanterelle, raw’, ‘Romaine lettuce, raw’, ‘Cabbage, raw’, ‘Celery root, raw’, ‘Oyster mushroom, raw’, ‘Tomato, unspecified, raw’, ‘Mushroom, raw’, ‘Alfalfa sprouts, raw’, ‘Leek, cooked’, ‘Onion, cooked’, ‘Cauliflower, peas, carrot, frozen’, ‘Stir-fry mix, Chinese type, stir-fried without fat’, ‘Stir-fry mix, Thai type, stir-fried without fat’, ‘Mushroom, fried in fat’, ‘Green pepper, cooked’, ‘Rutabaga, cooked’, ‘Eggplant, raw’, ‘Swiss chard, chard, raw’, ‘Chinese cabbage, raw’, ‘Radish, raw’, ‘Peas, carrot, frozen’, ‘Tomato, cooked’, ‘Field salad, raw’, ‘Corn, canned’. |
| Fruit and berries | ‘Banana, raw’, ‘Grape, blue, raw, without stone’, ‘Orange, raw, with peel’, ‘Nectarine, raw’, ‘Clementine, raw’, ‘Grape, green, raw, without stone’, ‘Apple, Red Aroma, raw’, ‘Avocado, raw’, ‘Apple, unspecified, raw’, ‘Pear, raw’, ‘Banana, raw, without peel’, ‘Raspberry, raw’, ‘Apple, imported, raw’, ‘Blueberry, raw, with peel’, ‘Grape, unspecified, raw, with stone’, ‘Watermelon, raw’, ‘Strawberry, raw’, ‘Grapefruit, raw’, ‘Lime, raw’, ‘Grape, green, raw, with stone’, ‘Mango, raw’, ‘Kiwi, raw’, ‘Sharon, persimmon, raw’, ‘Blackberry, raw’, ‘Lemon, raw’, ‘Apple, Granny Smith, Golden Delicious, raw’, ‘Passion fruit, raw’, ‘Pomegranate, raw’, ‘Cantaloupe melon, raw’, ‘Pineapple, raw’, ‘Cherry, raw’, ‘Currant, raw’, ‘Honeydew melon, raw’, ‘Passion fruit, raw’, ‘Jackfruit, raw’, ‘Plum, raw’, ‘Blueberry, American, raw’ |
| Vegetable products | ‘Green olives, pickles’, ‘Chopped tomatoes, tetra pack’, ‘Tomato paste, canned’, ‘Pickled cucumbers’, ‘Ketchup, tomato ketchup’, ‘Taco sauce’, ‘Pickled ginger’, ‘Sun-dried tomatoes, preserved in oil, drained’, ‘Tomato salsa’, ‘Water chestnuts, canned’, ‘Asparagus, canned, drained’, ‘Pickled beets’, ‘Guacamole’, ‘Nori, dried seaweed’, ‘Black olives, in oil, canned’, ‘Tomato paste’, ‘Green olives, stuffed with paprika’, ‘Crispy-fried onions’, ‘Sun-dried tomatoes, dried’, ‘Taco sauce, medium, Santa Maria’, ‘Taco sauce, medium, Old El Paso’, ‘Nori, dried seaweed’ |
| Fruits and berries products | ‘Mango salsa’, ‘Pineapple, canned, with own juice’, ‘Date, dried’, ‘Prunes’, ‘Raisins’, ‘Papaya, dried’, ‘Apple, dried’, ‘Strawberry, 100 g sugar per kg of berries, frozen’ |
| Legumes | ‘Lentils, green and brown, cooked with salt’, ‘Lentils, green and brown, dry’, ‘Lentils, red, cooked, canned’, ‘Lentils, red/pink, dry’, ‘Peas, green, cooked, canned’, ‘Kidney beans, red, canned’, ‘Black beans, canned’, ‘Edamame beans, soybeans, frozen, blanched’, ‘Tomato beans, white beans, in tomato sauce, cooked, canned’, ‘White beans, large, cooked, canned’, ‘Chickpeas, cooked, canned’, ‘Sugar peas, Norwegian, raw’, ‘Snap peas, frozen, blanched’, ‘Chickpea flour’, ‘Snap peas, frozen, blanched’, ‘Peas, green, cooked, canned’ |
| Nuts and seeds | ‘Chia seeds, dried’, ‘Flaxseeds, crushed’, ‘Peanut butter’, ‘Pistachios’, ‘Sesame seeds, without shell’, ‘Cashew nuts’, ‘Walnuts’, ‘Almonds’, ‘Salted cashew nuts’, ‘Hazelnuts’, ‘Squash seeds, pumpkin seeds’, ‘Sunflower seeds’, ‘Peanuts, salted’, ‘Peanuts, salted, Polly’, ‘Sesame seeds, with shell’, ‘Brazil nuts’, ‘Almond flour’ |
| Potatoes (including sweet potato) | ‘Stored potatoes, boiled with skin’, ‘Potato, early, with skin, boiled’, ‘Sweet potato, raw’, ‘Sweet potato, with skin, boiled without salt’, ‘Stored potatoes, boiled without skin, salted water’ |
| Milk and dairy products | ‘Crème Fraîche, 35% fat’, ‘Sour cream, light, 18% fat’, ‘Coffee cream, 10% fat’, ‘Whipping cream, 18% fat’, ‘Sour cream dressing, with herbs’, ‘Sour cream, light, 10% fat’, ‘Thick sour cream, 35% fat’, ‘Skimmed milk, 0.5-0.7% fat, vitamin D, unspecified’, ‘Chocolate milk’, ‘Cocoa, with skimmed milk, prepared’, ‘Low-fat milk, 1-1.2% fat, unspecified’, ‘Skimmed milk, Styrk’, ‘Skimmed milk, 0.5% fat, vitamin D, lactose-free’, ‘Whole milk, unspecified’, ‘Chocolate milk, powdered and skimmed milk’, ‘Low-fat milk, unspecified’, ‘Chocolate milk, lactose-free, no added sugar, Litago’, ‘Cultured milk, raspberry, Biola’, ‘Skimmed milk’, ‘Cultured milk, strawberry, Biola’, ‘Whole milk, 3.5% fat, Tine’, ‘Cultured milk, blueberry, lactose-free, Biola’, ‘Skyr, natural’, ‘Skyr, flavored, artificially sweetened’, ‘Yogurt, natural’, ‘Yogurt, Greek Lemon’, ‘Yogurt, with muesli and forest berries, Go'morgen’, ‘Yogurt, strawberry’, ‘Yogurt, vanilla, lactose-free’, ‘Quark, 1% fat, Kesam’, ‘Quark, 7% fat, Kesam’, ‘Milkshake, chocolate flavor’, ‘Chocolate milk, Styrk Cocoa’ (Norwegian name), ‘Whipping cream, 37% fat’, ‘YT Protein Milk Cocoa’, ‘Coffee whitener, powder, Coffeemate’, ‘Caffe latte, simple, with skimmed milk’, ‘Brie, white mold cheese’, ‘Østavind, yellow cheese’, ‘Ridder cheese’, ‘Gräddost’, ‘Gorgonzola, blue mold cheese’, ‘Jarlsberg, yellow cheese, light’, ‘Jarlsberg, yellow cheese’, ‘Mozzarella’, ‘Feta, goat milk cheese’, ‘Yellow cheese, unspecified’, ‘Cream whey cheese, brown cheese’, ‘Parmesan cheese’, ‘Norvegia, yellow cheese’, ‘Halloumi cheese’, ‘Norvegia, yellow cheese, rich’, ‘Gudbrandsdalsost, brown cheese’, ‘Cheddar cheese’, ‘Pizza cheese, grated’, ‘Edam cheese, yellow cheese’, ‘Ricotta cheese’, ‘Yellow cheese (Nøkkelost), light’, ‘Mozzarella, light’, ‘Lean cheese, spreadable, 10% fat’, ‘Cottage Cheese’, ‘Cream cheese, lean, Philadelphia’, ‘Lean cheese, spreadable, 7% fat’, ‘ Wheey cheese spread (Prim, Norwegian name), lean’, ‘Cream cheese, plain’, ‘Cream cheese, herb’, ‘Lean cheese, spreadable, 3% fat’, ‘Cream cheese, herbs’, ‘Bacon cheese, spreadable’ |
| Substitutes to dairy products | ‘Soy milk, fortified with calcium, unsweetened’, ‘Oat milk, fortified with calcium and vitamins’, ‘Rice milk’, ‘Vegetable cream substitute, whipping’, ‘Soy-based cream substitute, 17% fat’, ‘Almond milk’, ‘Vegetable-based cheese substitute’ |
| Substitutes to meat and vegetarian food products | ‘Vegetarian mince, soy protein, frozen’, ‘Falafel, chickpea balls, homemade’, ‘Tofu, soybean cheese’, ‘Soy burger, fried in vegetable oil’, ‘Soy sausage, canned’, ‘Hummus’, ‘Sesame paste, tahini’, ‘Vegetable pâté, Tartex’, ‘Vegetarian burger, homemade’, ‘Bean patties, fried in soy oil’ |
| Vegetarian dishes | ‘Sushi, maki, mango, vegetarian’, ‘Vegetarian lasagna’, ‘Bean stew, vegetarian’, ‘Stew base, with rice, Mexican, meatless’, ‘Sushi, maki, mango, vegetarian’, ‘Salad, Greek, with feta cheese, olives’, ‘Carrot soup, homemade’, ‘Cauliflower soup, from powder, prepared’, ‘Cauliflower/broccoli soup, Ready in a cup, prepared’, ‘Tomato soup, with macaroni, from powder, prepared’, ‘Sweet potato soup, homemade’, ‘Minestrone soup, homemade’, ‘Rutabaga mash’, ‘Pasta, fresh with cheese, ravioli, cooked’, ‘Hash browns, fried potatoes and onions, frozen, bought’ |
| Red meat (including processed meat) | ‘Beef, sirloin steak, fried’, ‘Pork, sirloin, fried’, ‘Pork, roast without fat edge, oven-roasted’, ‘Pork, family rib roast, fried’, ‘Game meat, roast, oven-roasted’, ‘Beef, tenderloin, eaten raw’, ‘Lamb, chop, fried’, ‘Pinnekjøtt (cured lamb ribs), boiled, with bones’, ‘Roast beef, slices, cold cuts’,‘Grill sausage’, ‘Meat sausage, smoked/unsmoked’, ‘Chorizo sausage’, ‘Meat sausage, with less fat’, ‘Ground beef, fried without fat’, ‘Minced beef, beef, fried without fat’, ‘Pork shoulder’, ‘Liver pâté, baked in the oven’, ‘Liver pâté, canned’, ‘Meat patties’, ‘Meatballs’, ‘Cured ham’, ‘Salami’, ‘Salami, lean, Gilde’, ‘Meatballs, fried’, ‘Salami, Grilstad jubilee salami (Norwegian name)’. ‘Cured ham, Strandaskinke, Grilstad (Norwegian name)’, ‘Liver pâté, baked in the oven, Vita’, ‘Liver pâté, baked in the oven, Mills’, ‘Turkey and chicken grill sausage’, ‘Bologna, lean, Gilde’, ‘Salami, Danish, First Price’, ‘Meat sausage, Gilde’, ‘Meatballs, fried’, ‘Pork, bacon with rind, fried’, ‘Boiled ham’, ‘Boiled ham, Nordfjord’, ‘Smoked pork shoulder’ |
| White meat (including processed meat) | ‘Chicken, fillet, fried in fat’, ‘Chicken, thigh, upper thigh, and drumstick, without skin, oven-roasted’, ‘Chicken, fillet, boiled’, ‘Turkey fillet, natural/smoked’, ‘Ground chicken, fried without fat’, ‘Turkey roll, spiced ham’ |
| Eggs, all types | ‘Egg, boiled, without shell’, ‘Egg, fried in fat’, ‘Scrambled eggs, fried in fat’, ‘Scrambled eggs, fried without fat’, ‘Egg, raw, without shell’, ‘Scrambled eggs, fried without fat’, ‘Organic egg, fried’, ‘Omelette, fried without fat’, ‘Omelette, fried in fat’ |
| Fish and shellfish | ‘Salmon, fillet, gravlax’, ‘Trout, farmed, boiled, with skin and bones’, ‘Salmon, fillet, pan-fried’, ‘Salmon, smoked, fillet’, ‘Mackerel, July-September, fatty, pan-fried, fillet’, ‘Salmon, fillet, oven-roasted’, ‘Sashimi, salmon’, ‘Cod, fillet, boiled’, ‘Cod, fillet, breadcrumb-coated, fried in fat’, ‘Perch, without skin and bones, boiled’, ‘Cod, fillet, oven-roasted’, ‘Cod, breaded, pre-fried, oven-roasted without fat’, ‘Shrimps, boiled’, ‘King prawn, boiled, no shell‘, ‘peeled shrimps in brine’ |
| Fish products | ‘Kaviar mix’, ‘Fish cakes, Lofoten’, ‘Mackerel fillet, in tomato sauce, 60% mackerel, canned’, ‘Tuna, in oil, not drained, canned’, ‘Fish balls, drained, canned’, ‘Tuna, in water, drained, canned’, ‘Caviar, from cod roe’, ‘Caviar, pollock roe’, ‘Fish cakes, purchased’, ‘Mackerel fillet, in tomato sauce, 70% mackerel, canned’, ‘Sushi, nigiri, halibut’  ‘Sushi, maki, California’, ‘Fish stew, with cod and tomato’, ‘Fish gratin, purchased’, ‘Fish fingers, pre-fried, fried in fat’, ‘Sushi, maki, tempura’, ‘Sushi, nigiri, salmon’, ‘Caviar, from capelin roe’, ‘Fish patties, Fiskemannen (Norwegian brand)’, ‘Mackerel fillet, in tomato sauce, 50% mackerel, in a container’, ‘Sushi, maki, tempura’, ‘Fish fingers, pre-fried, fried in butter’, ‘Fish patties, lean fish’, ‘Fish soup, Bergen style, from powder, prepared’, ‘Fish gratin, with egg and macaroni, oven-baked’ |
| Convenience food | ‘Pizza, with tomato sauce and cheese’, ‘Store-bought pizza’, ‘Homemade ham pizza’, ‘Homemade Parma pizza’, ‘Pizza, with tomato sauce and cheese’, ‘Grandiosa Original pizza’, ‘Homemade vegetarian pizza’, ‘Ristorante Mozzarella pizza’, ‘Homemade minced meat pizza’, ‘Big One Classic pizza’, ‘Fried chicken nuggets, fast food’, ‘Cheeseburger, with bun, dressing, etc., store-bought’, ‘Pizza swirls’, ‘Grandiosa minced meat and onion pizza’, ‘Simple hamburger, with bun, dressing, etc., store-bought’, ‘Chicken burger, with bun, dressing, etc., store-bought’, ‘French fries, purchased, oven-baked’, ‘French fries, takeout’, ‘Instant mashed potatoes, with milk’, ‘Potato salad, with sour cream’, ‘Potato flour’ |
| Dessert, cake, and sweets | ‘Donuts, with chocolate glaze, purchased’, ‘Sheet cake, with frosting, purchased’, ‘Brownies, with chocolate glaze, purchased’, ‘Sveler (Norwegian), pancakes’, ‘Apple cake’, ‘Muffins’, ‘Spice cake’, ‘Twist doughnuts’, ‘Cheesecake, with cookie crust’, ‘Muffins, with blueberries, purchased’, ‘Carrot cake, with cream cheese frosting, purchased’  ‘Pavlova, with whipped cream and berries’, ‘Pancakes, with skim milk, cooked in soft margarine’, ‘Muesli bar, energy bar, homemade’, ‘Cookies, sweet, chocolate chip, large’  ‘White gingerbread men’, ‘Cookies, sweet, oat, Bixit’, ‘Ginger snaps’, ‘Cookies, sweet, Gjende (Norwegian brand name)’, ‘Cookies, sweet, oat flakes’, ‘Bun, without raisins, purchased’, ‘School bread, with powdered sugar glaze, custard, coconut, purchased’, ‘Cinnamon bun, with sugar sprinkles, purchased’, ‘Danish pastry, with custard’, ‘Bun, with chocolate chips’, ‘Viennese pretzel, with almond filling’, ‘Croissant, without filling, purchased’, ‘Berliner, purchased’, ‘Waffles, with egg and whole milk’, ‘Snickers cake’, ‘Marzipan cake, with jam and cream’, ‘Banana cake’, ‘Pancakes, with banana’, ‘Cookies, sweet, chocolate chip, nuts, small’, ‘Cookies, sweet, children's cookies with chocolate’, ‘Cookies, sweet, oat and chocolate’, ‘Cookies, sweet, waffle, filled’, ‘Brown sticks’, ‘Cookies, sweet, whole grain, Digestive’, ‘Raisin bun, purchased’, ‘Waffles, from powder, Toro’, ‘Thick lefse, sandwich, purchased’, ‘Christmas cake’, ‘Waffles, coarse’, ‘traditional Norwegian flat bread with sweetened filling (Vestlandslefsa, Norwegian brand name)’, ‘Pancakes, with whole milk, cooked in hard margarine’, ‘Kringle, yeast dough, with dried fruit’, ‘Pepper nuts’, ‘Biscotti’, ‘Waffles, with egg, skim milk’, ‘Chocolate pudding, Piano’, ‘Vanilla sauce, Piano’, ‘Caramel pudding, Piano’, ‘Ice cream, cream-based’, ‘Ice cream, ice cream stick, Royal Dark’, ‘Caramel sauce’, ‘Sorbet’, ‘Fruit ice’, ‘Whipped cream, canned’, ‘Chocolate fondant’, ‘Ice cream, light ice cream, with less sugar and fat’, ‘Rice pudding with strawberries’, ‘YT Protein Pudding Chocolate’, ‘YT Recovery Bar Chocolate/Peanut’, ‘Jelly, from powder, prepared’, ‘Vanilla sauce, Piano’, ‘Almond ring cake’, ‘Layerd apple dessert with crumbs (Tilslørte bondepiker in Norwegian)’, ‘Sugar, white’, ‘Syrup’, ‘Vanilla sugar’, ‘Honey’, ‘Sugar, brown’, ‘Chocolate, Mars’, ‘Mixed candy, without chocolate’, ‘Candy, drops’, ‘Chocolate, Snickers’, ‘Chocolate, with jelly and marzipan, Troika’, ‘Chocolate, dark, 70% cocoa’, ‘Chocolate, with biscuits’, ‘Chocolate, cooking chocolate, dark’, ‘Chocolate, with coconut filling, Bounty’, ‘Jelly candy, Jellymen’, ‘Chocolate, small chocolates’, ‘Chocolate, with nuts’, ‘Chocolate, milk chocolate’, ‘Chocolate, filled, confectionery’, ‘Caramel, with chocolate coating’, ‘Marshmallow, marshmallows’, ‘Caramel’, ‘Licorice, sweet’, ‘Chocolate, milk chocolate, porous’, ‘Mixed candy, salty’, ‘Marzipan bread, with chocolate coating’, ‘Chocolate, with mint filling, After Eight’, ‘Marzipan, 30% almonds’ |
| Sweetened bread spread | ‘Hazelnut spread, Nugatti’, ‘Milk spread, with sugar, Hapå (Norwegian brand name)’, ‘Jam, 60% berries, no added sugar’, ‘Jam, 45% berries, 25% sugar’, ‘Marmalade, 25% orange, 65% sugar’, ‘Jam, 40% berries, 40% sugar’, ‘Jam, 60% berries, 30% sugar’ |
| Sweetened cereal | ‘Oat rings, Nestlé Cheerios’, ‘Cereal mix, muesli with fruit, nuts, sweetened’, ‘Breakfast cereal, chocolate flavor’, ‘Cereal mix, muesli with fruit, nuts, sweetened’, ‘Cereal mix, with berries, Go'dag Blueberry Muesli’, ‘Breakfast cereal, chocolate flavor, Coco Pops Crunchers’, ‘Cereal mix, 4-grain’, ‘Roasted oats, with fruit, oil, Crüsli’, ‘Wheat biscuits, Weetabix’, ‘Cereal mix, with fruit, AXA Fruit Muesli’, ‘Oat flakes’ |
| Salted snacks | ‘Cookies, salty, fatty, Ritz, Seltiner’, ‘Chili nuts’, ‘Potato chips’, ‘Potato chips, with less fat’, ‘Popcorn, purchased’, ‘Tortilla chips, nacho chips, Maarud Tortilla Cheese’  ‘Tortilla chips, nacho chips’, ‘Popcorn, microwave’, ‘Potato chips, Kims’, ‘Cheese puffs’, ‘Salt sticks’, ‘Popcorn, popped in soybean oil’, ‘Potato chips, Maarud’, ‘Taco shells’, ‘Nut mix, oriental blend’ |
| Alcoholic beverages | ‘White wine, dry, 12% alcohol by volume’, ‘Lager, 4.7% alcohol by volume’, ‘Beer, reduced energy content, low carb’, ‘White wine, semi-dry, 10% alcohol by volume’  ‘Red wine, 12% alcohol by volume’, ‘Cider, sweet, 4.5% alcohol by volume’, ‘Liqueur, 17% alcohol by volume, cream-based’, ‘White wine, dry, 12% alcohol by volume’, ‘White wine, rosé, sparkling’ |
| Non-sugary drinks (including energy drinks) | ‘Soft drink, artificially sweetened, ready to drink’, ‘Soda, cola, artificially sweetened, light’, ‘Iced tea, artificially sweetened, light, Nestea’, ‘Energy drink, added taurine, caffeine, and B-vitamins, sugar-free’ |
| Juice and smoothie | ‘Smoothie, pineapple and mango’, ‘Orange juice’, ‘Apple juice, freshly pressed’, ‘Smoothie, with yogurt, juice, banana, and berries’, ‘Smoothie, blueberry and apple’, ‘Smoothie, raspberry and strawberry’, ‘Grapefruit juice’, ‘Smoothie, blueberry and pomegranate’, ‘Tropical juice’, ‘Lemon juice, bottle’, ‘Smoothie, with juice, banana, and berries’ |
| Sugar-sweetened beverages (including energy drinks) | ‘Household juice, ready to drink’, ‘Soda, cola, with sugar’, ‘Soda, fruit juice, with sugar, Solo, Mozell, etc.’, ‘Blackcurrant juice, 1000 g sugar per liter raw juice, ready to drink’, ‘Blackcurrant syrup, ready to drink’, ‘Blackcurrant syrup, undiluted’, ‘Blackcurrant juice, 500 g sugar per liter raw juice, ready to drink’, ‘Iced tea, Nestea’, ‘Apple nectar’, ‘Caffe mocha’, ‘Energy drink, added taurine, caffeine, and B-vitamins’ |
| Vegetable oils | ‘‘Olive oil’, ‘Sunflower oil’, ‘Olive oil, extra virgin’, ‘Coconut oil’, ‘Canola oil’, ‘Vita Hjertego' oil’, ‘Canola and sunflower oil, Melange’, ‘Frying oil’, ‘Coconut oil’, ‘Sesame oil’, ‘Ghee, vegetable’. |
| Butter/margarine | Butter’, ‘Margarine, soft’, ‘Bremykt (Norwegian brand name)’, ‘Margarine, Vita Hjertego (Norwegian brand name)', ‘Margarine, frying margarine’, ‘Brelett’, ‘Margarine, Melange’, ‘Margarine, Vita Hjertego' light’, ‘Butter, dairy butter, extra salt, Kviteseidsmør’, ‘Margarine, Melange, without salt and milk, foil’, ‘Margarine, Soft Flora light’, ‘Margarine, Soft Flora, original’ |
|  | **Food groups not included in the tables in the main manuscript** |
| Mixed dish with meat | ‘Lasagna, with minced meat’, ‘Lasagna, with minced meat, frozen, bought’, ‘Beef stroganoff’, ‘Lamb stew (Fårikål)’, ‘Taco with tortilla, minced meat, vegetables’, ‘Stew, light, beef’’, ‘Taco with tortilla, chicken, vegetables’, ‘Chili con carne’, ‘Pasta Bolognese, homemade’ |
| Water, coffee and tea | ‘Water, drinking water’, ‘Mineral water, carbonated, Bris naturell’, ‘Mineral water, carbonated, Farris naturell’, ‘Mineral water, carbonated, Bonaqua naturell’, ‘Coffee, brewed, boiled’, ‘Tea, green, prepared’, ‘Coffee, instant, prepared’, ‘Espresso, single’, ‘Tea, black, prepared’ |
| Dressing/sauce | ‘Mayonnaise, light, 40% fat’, ‘Caesar dressing’, ‘Aioli’, ‘Dressing, mayonnaise-type, 55% fat’, ‘Mayonnaise, genuine’, ‘Mustard sauce’, ‘Salad dressing, oil and vinegar, French dressing’, ‘Soy sauce’, ‘Brown sauce, with onions, from powder’, ‘Pesto, green, store-bought’, ‘Dipping sauce, sweet and sour barbecue sauce’, ‘Bearnaise sauce, from powder, prepared’, ‘Pesto, green, homemade’, ‘Brown sauce, meatball sauce, from powder, prepared’, ‘White sauce, from powder, prepared’, ‘Bearnaise sauce, from powder, prepared’, ‘Sandefjord butter’, ‘Dressing, mayonnaise-type, 30% fat’, ‘Hollandaise sauce’, ‘Shrimp salad, mayonnaise salad, 35% fat’, ‘Italian salad, mayonnaise salad, 37% fat’, ‘Tzatziki’ |
