# Supplementary material for: Food groups, macronutrient intake and objective measures of total carotenoids and fatty acids in 16-to-24-year-olds following different plant-based diets compared to an omnivorous diet
Source: PLoS One. 2025 Jan 17;20(1):e0311118. doi: 10.1371/journal.pone.0311118 (PMC11741618; doi:10.1371/journal.pone.0311118)
Supplement: S2 Table — (DOCX) [file pone.0311118.s002.docx]

**Supplemental Table 2. Changes made to food subcategories available in the Norwegian version of Myfood24.**

| ***Original food category*** | **Original food subcategories** | **Changes made to the food subcategories available in the Norwegian version of Myfood24** |
| --- | --- | --- |
| ‘Grains, baked goods, and nuts’ | Grains, rice, pasta, raw materials Grains, rice, pasta, cooked Crackers, crispbreads, etc. Porridge  Cereal mix, breakfast cereals  Bakery products, homemade  Bakery products industrially baked.  Nuts, almonds, and seeds | ‘Grains, rice, pasta, raw’ ‘materials Grains, ‘rice, pasta, cooked’, ‘crispbreads, etc.’ ‘Porridge Cereal mix,’ ‘Bakery products, homemade’, ‘Bakery products, industrially baked’ were split into ‘refined grain products’ and whole grain products’.  ‘Breakfast cereals’ was split into ‘sweetened cereals (new food subcategory)’ and into the food subcategories ‘refined grain products’, ‘whole grain products’.  Crackers were split into the food subcategories ‘salted snacks’ (new food subcategory) and the food subcategory ‘refined grain’ depending on the brand and product.  Nuts, almonds, and seeds – no changes |
| Snacks | Snacks  Cookies, sweet biscuits, breadcrumbs  Sweet yeast pastries, flatbreads, waffles, etc.  Other pastries, etc.  Chocolate and other confectionery Desserts, ice cream, and sweets  Sugar, honey, and sweet spreads | The food subcategory ‘snacks’ were slightly altered to consist of ‘salted snacks’ only. Not salted snacks were entered into relevant food subgroups such as ‘refined grain products’.  The original food subcategories ‘sugar’ ‘Cookies, sweet biscuits, breadcrumbs’, ‘Sweet yeast pastries, flatbreads, waffles, etc’.  ‘Other pastries, etc.’, ‘Chocolate and other confectionery Desserts, ice cream, and sweets’ were combined into one food sub category ‘desserts, cakes, and sweets’ (**See supplemental table 1**)    The subcategory ‘sweet spreads’ were made as a separate food subcategory ‘sweetened bread spreads’ such as sweetened fruit products as marmalade and jam were added (full description in S**upplemental table 1**). |
| Fruits, vegetables, and legumes | Fruits and berries products  Fruits and berries, raw/fresh  Legumes  Vegetables, cooked  Vegetables, raw and frozen    Vegetable products, for example,  Potatoes | Fruits and berries products – marmalade and jam were removed from the original food subcategory and entered into the new food subcategory ‘sweetened bread spread’  Fruits and berries, raw/fresh – no changes made.  Legumes – no changes made.  ‘Vegetables, cooked’ and ‘Vegetables, raw and frozen’ were combined into one new food subcategory ‘vegetables’. In addition, mixed frozen vegetables were removed from the food subcategory ‘vegetable products’ and entered into the subcategory ‘vegetable’.  Vegetable products – the food items vegetable mix and canned corn were removed from ‘vegetable products’ and entered into the subcategory ‘vegetables’. The food items based on ‘fried potatoes’ were removed and entered into a new food subcategory ‘convenience food’. The potato food items (including sweet potato) (see **supplemental table 1**) were removed and entered into a new food subcategory ‘potatoes’ (variable of relevance for Nordic setting). |
| Ingrediens | Flour  Herbs and spices | Flour was split into the food subcategories ‘refined grain’ or ‘nuts and seeds’ (e.g. almond flour) depending on the reported type of flour.  Herbs and spices – were not entered into any of the subcategories. |
| Fats | Margarine and butter Mayonnaise, dressings, etc. Cooking oil, frying fat, etc. Various ingredients, for example, sea salt, mustard | Margarine and butter – split into a new separate food subcategory ‘butter/margarine’  Mayonnaise, dressings, etc. – split into a new separate food subcategory ‘dressing/sauce’  Cooking oil, frying fat, etc. – split into a new separate food subcategory ‘vegetable oil’  Various ingredients, for example, sea salt, mustard – ingredients as sea salt were not entered into any new subcategory, and ingredients as mustard were entered into the subcategory ‘dressing/sauce’ |
| Ready meals | Vegetarian products and dishes  Soups, sauces, and stew bases  Soup powder  Pizza, pie, tacos, etc. | Vegetarian products and dishes – ‘vegetarian products’ were split into new food subcategories ‘dairy substitutes’ and ‘meat substitutes’. ‘Vegetarian dishes’ were split into a new separate food subcategory named ‘vegetarian dishes’  Soups, sauces, and stew bases – vegetarian soups were removed and entered into the new subcategory ‘vegetarian dishes’ and soup containing fish were removed and entered into the new subcategory ‘fish products’. ‘Sauces’ were removed and entered into the new subcategory ‘dressing/sauces’. ‘Stew bases’ were entered into the new subcategory vegetarian dishes if vegetarian, the new subcategory ‘dish with meat’ if containing meat (all types) or ‘fish products’ if containing fish.  Soup powder – was entered into ‘vegetarian dishes’ if e.g. tomato soup powder or cauliflower powder.  Pizza, pie, tacos, etc. – food items were split and entered into the new subcategories ‘convenience foods’ and ‘meat dishes’ (see **supplemental table 1** for food items included in the different subcategories). Vegetarian pizza was not categoriez as ‘vegetarian dish’ but also as ‘convenience food’ for these food items to be placed equally for the dietary groups. |
| Meat and meat products | Dishes with poultry and meat  Beef, veal, cooked  Pork, cooked  Poultry, cooked  Various meats, minced meat, offal, cooked  Meat products and cold cuts  Sausages | Dishes with poultry and meat – entered into the new subcategory ‘meat dishes’  Beef, veal, cooked – entered into the new subcategory ‘red meat’  Pork, cooked - entered into the new subcategory ‘red meat’  Poultry, cooked - entered into the new subcategory ‘white meat’  Various meats, minced meat, offal, cooked - – split into a new subcategory ‘processed meat products’, if not processed meat, the food item was placed in the new subcategories ‘red meat’ or ‘white meat’, see **supplemental table 1** for food items in the different subcategories.  Meat products and cold cuts – split into a new subcategory ‘processed meat products’  Sausages – split into a new subcategory ‘processed meat products’ |
| Dairy products | Cheese, full-fat  Cheese, semi-fat, lean  Cheese, extra fat  Milk and milk-based drinks  Cream, sour cream, cream substitute | No changes made |
| Fish and seafood | Dishes with fish, seafood, etc.  Fatty fish, cooked  Lean fish, cooked  Fish products and fish spreads | ‘Dishes with fish’, ‘fish products’ and ‘fish spread’ – were entered into a new subcategory ‘fish products’  ‘Seafood’, ‘Fatty fish, cooked’ ‘Lean fish, cooked’ – were entered into a new subcategory ‘lean and fatty fish and seafood’ |
| Eggs | Eggs (all types) | No changes made |
| Beverages | Juice, soda, etc.  Alcoholic beverages  Water, coffee, tea | Juice, soda, etc. – split into three new subcategories ‘sugary beverage’, ‘non-sugary beverage’ and ‘juice and smoothie’ (see **supplemental table 1** for included food items). In addition, the food items for plant-based drinks were removed and entered into dairy substitutes.  Alcoholic beverages – split into a separate subcategory ‘alcoholic drinks’  Water, coffee, tea – splint into a separate subcategory ‘water, coffee, tea’ |
