# Supplementary material for: Food groups, macronutrient intake and objective measures of total carotenoids and fatty acids in 16-to-24-year-olds following different plant-based diets compared to an omnivorous diet
Source: PLoS One. 2025 Jan 17;20(1):e0311118. doi: 10.1371/journal.pone.0311118 (PMC11741618; doi:10.1371/journal.pone.0311118)
Supplement: S3 Table — (DOCX) [file pone.0311118.s003.docx]

**Supplemental Table 3. Food items included in the carotenoid-rich food categories in VeggiSkills-Norway.**

|  | **Food items included in the carotenoid-rich food categories** |
| --- | --- |
| ß-carotene-rich foods | **Carrot**: ‘Carrot, raw’, ‘Carrot, cooked’, ‘Carrot soup’ (not included in carrot: ‘Cauliflower, peas, carrot, frozen’, Peas, carrot, frozen’, ‘Carrot cake, with cream cheese frosting, purchased’); **Broccoli**: ‘Broccoli, raw’, ‘Broccoli, frozen, blanched’, ‘Broccoli, cooked’ (not included in broccoli: ‘Cauliflower/broccoli soup, Ready in a cup, prepared’; **Pepper:** ‘Red pepper, raw’, ‘Red pepper, cooked’, ‘Green pepper, cooked’, Yellow pepper, raw’; **Lettuce**: ‘Lettuce, raw, ’Iceberg lettuce, raw’, ‘Romaine lettuce, raw’, arugula; **Leek**: ‘Leek, raw’, ‘Leek cooked’; **Spinach**: ‘Spinach, raw’, ‘Spinach, frozen’; **Cantaloupe melon**: ‘Cantaloupe melon, raw’; **Parsley**: ‘Parsley, cooked’; **Chili**: ‘Chili, red, raw’, ‘chili, powder’; **Driet apricot and parsley**: Not reported. |
| α-carotene-rich foods | Carrot: ‘Carrot, Norwegian, raw’, ‘Carrot, cooked’, ‘Carrot soup, homemade’ (not included in carrot): ‘Cauliflower, peas, carrot, frozen’, Peas, carrot, frozen’, ‘Carrot cake, with cream cheese frosting, purchased’ |
| ß-cryptoxanthin-rich foods | **Orange juice**: ‘Orange juice’; **Clementine**: ‘Clementine, raw’; **Pepper**: ‘Red pepper, raw’, ‘Red pepper, cooked’, ‘Green pepper, cooked’, Yellow pepper, raw’; **Orange**: ‘Orange, raw’; **Corn**: ‘Canned corn’; **Mango:** ‘Mango, raw’; **Watermelon**: ‘Watermelon, raw’; **Popcorn**: ‘Popcorn, bought’, popcorn, microwave’, ‘popcorn, popped in soy oil’; **Chili**: ‘Chili, red, raw’, ‘chili, powder’; **Pineapple**: ‘Pineapple, raw’, Canned pineapple’; **Peach and basil**: not reported. |
| lycopene-rich foods | **Canned tomato**: ‘Canned tomatoes’; **Fresh tomato**: ‘Tomato, imported, raw’: **Ketchup**: ‘ketchup, tomato ketchup’; **Fresh cherry tomato**: ‘Cherry tomato, raw’**; Tomato puree**: Canned tomato puree’; **Watermelon**: ‘Watermelon, raw’; **Boiled tomato**: ‘Tomato soup with macaroni, powder, prepared’; **Tomato soup powder**: ‘Tomato soup, powder’ |
| lutein+zeaxanthin-rich foods | **Broccoli**: ‘Broccoli, raw’, ‘Broccoli, frozen, blanched’, ‘Broccoli, cooked’ (not included in broccoli: ‘Cauliflower/broccoli soup, Ready in a cup, prepared’; **Pepper**: ‘Red pepper, raw’, ‘Red pepper, cooked’, ‘Green pepper, cooked’, Yellow pepper, raw’; **Lettuce**: ‘Lettuce, raw’ (Norwegian; bladsalat, feltsalat), ’Iceberg lettuce, raw’, ‘Romaine lettuce, raw’, arugula; **Leek**: ‘Leek, raw’, ‘Leek cooked’; **Spinach**: ‘Spinach, raw’, ‘Spinach, frozen’; **Parsley**: ‘Parsley, cooked’; **Corn**: ‘Canned corn’; **Corn meal**: ‘Tortilla, cornflakes’; **Popcorn**: ‘Popcorn, bought’, popcorn, microwave’, ‘popcorn, popped in soy oil’; **Peas**: ‘Canned peas, green, cooked’; **Corn flour**: ‘corn starch’; **Brussels sprouts**: ‘Brussel sprouts, frozen’; **Basil and cornflakes** not reported. |
| total carotenoid-rich foods | All carotenoid-rich food groups combined into a total carotenoid-rich food variable |

The categorization into the carotenoid-rich food categories is based on a previous validation paper: Medin AC, Carlsen MH Andersen LF (2016) Associations between reported intakes of carotenoid-rich foods and concentrations of carotenoids in plasma: a validation study of a web-based food recall for children and adolescents. *Public Health Nutrition* 19(18), 3265-3275.
