# Supplementary material for: Food groups, macronutrient intake and objective measures of total carotenoids and fatty acids in 16-to-24-year-olds following different plant-based diets compared to an omnivorous diet
Source: PLoS One. 2025 Jan 17;20(1):e0311118. doi: 10.1371/journal.pone.0311118 (PMC11741618; doi:10.1371/journal.pone.0311118)
Supplement: S4 Table — (DOCX) [file pone.0311118.s004.docx]

**Supplemental Table 4. Median absolute food group intake among Norwegian youth with different dietary practice**

| **Food groups, g/d** | **All**  **n = 165** | | **Vegans**  **n = 19** | | **Lacto-ovo-vegetarians**  **n = 20** | | **Pescatarians**  **n = 30** | | **Flexitarians**  **n = 25** | | **Omnivores**  **n = 71** | | **P-value** |
| --- | --- | --- | --- | --- | --- | --- | --- | --- | --- | --- | --- | --- | --- |
| **Plant-sourced foods** | **Median** | **25p,75p** | **Median** | **25p,75p** | **Median** | **25p,75p** | **Median** | **25p,75p** | **Median** | **25p,75p** | **Median** | **25p,75p** |  |
| Whole grain products, g/d^‡^ | 74 | 37, 128 | 82 | 43, 147 | 78 | 16, 123 | 80 | 60, 131 | 75 | 54, 126 | 60 | 18, 135 | 0.57 |
| Refined grain products, g/d^‡^ | 54 | 25, 100 | 100 | 32, 131 | 48 | 21, 77 | 76 | 35, 102 | 38 | 17, 90 | 53 | 24, 100 | 0.20 |
| Vegetables (all types), g/d^‡^ | 88 | 48, 134 | 137^*^ | 101, 223 | 82^†^ | 36, 121 | 95 | 73, 161 | 84 | 58, 121 | 65^†^ | 23, 124 | **<0.001** |
| Fruit and berries (not including juice/smoothie), g/d^‡^ | 123 | 30, 235 | 236 | 65, 350 | 120 | 55, 227 | 88 | 0, 229 | 137 | 81, 255 | 119 | 0, 187 | 0.07 |
| Legumes, g/d^‡^ | 0 | 0, 24 | 32^*^ | 7, 85 | 23^*,†^ | 0, 47 | 0 | 0, 39 | 0^†^ | 0, 19 | 0^§^ | 0, 0 | **<0.001** |
| Nuts and seeds, g/d^‡^ | 0 | 0, 6 | 28^*^ | 0, 55 | 0^†^ | 0, 3 | 0^†^ | 0, 3 | 0^†^ | 0, 9 | 0^†^ | 0, 0 | **<0.001** |
| Vegetable oil, g/d^‡^ | 0 | 0, 3 | 2^*^ | 0, 10 | 0 | 0, 5 | 0 | 0, 3 | 0 | 0, 2 | 0^†^ | 0, 1 | **0.034** |
| Potatoes (including sweet potatoes), g/d^‡│^ | 0 | 0, 30 | 0 | 0, 23 | 0 | 0, 12 | 0 | 0, 32 | 9 | 0, 32 | 0 | 0, 39 | 0.58 |
| Vegetable products, g/d^‡^ | 3 | 0, 18 | 5 | 3, 36 | 7 | 0, 12 | 9 | 0, 20 | 2 | 0, 17 | 1 | 0, 10 | 0.18 |
| Fruit and berry products, g/d^‡^ | 0 | 0, 0 | 0^*^ | 0, 16 | 0 | 0, 0 | 0^†^ | 0, 0 | 0 | 0, 0 | 0^†^ | 0, 0 | **0.009** |
| Dairy product substitutes, g/d^‡^ | 0 | 0, 6 | 60^*^ | 8, 125 | 0^†, a^ | 0, 9 | 0^†, a^ | 0, 4 | 0^†^ | 0, 50 | 0^§,a^ | 0, 0 | **<0.001** |
| Meat substitutes and vegetarian food products, g/d^‡¶^ | 0 | 0, 15 | 51^*^ | 15, 90 | 13^*^ | 0, 30 | 0^†^ | 0, 38 | 0^†^ | 0, 4 | 0^§^ | 0, 0 | **<0.001** |
| Vegetarian dishes, g/d^‡^ | 0 | 0, 0 | 0 | 0, 0 | 0 | 0, 74 | 0 | 0, 0 | 0 | 0, 24 | 0 | 0, 0 | 0.17 |
| **Animal-sourced foods** |  |  |  |  |  |  |  |  |  |  |  |  |  |
| Milk and dairy products (including cheese), g/d^‡^ | 120 | 18, 229 | 0 | 0, 0 | 60 | 29, 222 | 126 | 31, 230 | 139 | 62, 198 | 159 | 53, 299 | **̶** |
| Eggs (all types), g/d^‡^ | 0 | 0, 42 | 0 | 0, 0 | 18 | 0, 50 | 0 | 0, 49 | 10 | 0, 45 | 12 | 0, 48 | **̶** |
| Red meat (all types), g/d^‡^ | 0 | 0, 19 | 0 | 0, 0 | 0 | 0, 0 | 0 | 0, 0 | 0 | 0, 23 | 18 | 2, 62 | **̶** |
| White meat (all types), g/d^‡^ | 0 | 0, 6 | 0 | 0, 0 | 0 | 0, 0 | 0 | 0, 0 | 0 | 0, 9 | 0 | 0, 38 | **̶** |
| Lean, fatty fish and shellfish, g/d^‡^ | 0 | 0, 47 | 0 | 0, 0 | 0 | 0, 0 | 0 | 0, 72 | 10 | 0, 98 | 0 | 0, 63 | **̶** |
| Fish products, g/d^‡^ | 0 | 0, 10 | 0 | 0, 0 | 0 | 0, 0 | 0 | 0, 40 | 0 | 0, 23 | 0 | 0, 31 | **̶** |
| Butter/margarine, g/d^‡^ | 2 | 0, 6 | 0 | 0, 0 | 2 | 0, 4 | 4 | 0, 10 | 2 | 0, 4 | 3 | 0, 6 | **̶** |
| **Sugary, salted and convenience foods** |  |  |  |  |  |  |  |  |  |  |  |  |  |
| Dessert, cake, and sweets, g/d^‡^ | 35 | 9, 83 | 11^*^ | 0, 30 | 34 | 14, 100 | 56^†^ | 18, 100 | 43 | 16, 72 | 35 | 5, 92 | **0.019** |
| Sweetened bread spread, g/d^‡^ | 0 | 0, 5 | 0 | 0, 9 | 0 | 0, 5 | 0 | 0, 9 | 0 | 0, 7 | 0 | 0, 4 | 0.76 |
| Sweetened cereal, g/d^‡^ | 0 | 0, 1 | 0 | 0, 0 | 0 | 0, 16 | 0 | 0, 0 | 0 | 0, 9 | 0 | 0, 10 | 0.39 |
| Salted snacks, g/d^‡^ | 0 | 0, 10 | 0 | 0, 13 | 1 | 0, 10 | 0 | 0, 13 | 0 | 0, 8 | 0 | 0, 8 | 0.82 |
| Convenience foods, g/d^‡^ | 0 | 0, 100 | 0^*^ | 0, 0 | 6 | 0, 75 | 0 | 0, 105 | 0 | 0, 63 | 42^†^ | 0, 150 | **0.014** |
| **Beverages** |  |  |  |  |  |  |  |  |  |  |  |  |  |
| Alcoholic beverages, g/d^‡^ | 0 | 0, 0 | 0 | 0, 0 | 0 | 0, 0 | 0 | 0, 0 | 0 | 0, 16 | 0 | 0, 0 | 0.74 |
| Non-sugary beverages, g/d^‡^ | 0 | 0, 164 | 0 | 0, 133 | 0 | 0, 28 | 25 | 0, 202 | 0 | 0, 0 | 0 | 0, 200 | 0.13 |
| Juice and smoothie, g/d^‡^ | 0 | 0, 75 | 38 | 0, 100 | 0 | 0, 133 | 0 | 0, 67 | 38 | 0, 71 | 0 | 0, 80 | 0.80 |
| Sugar-sweetened beverages, g/d^‡^ | 0 | 0, 85 | 0 | 0, 0 | 0 | 0, 76 | 0 | 0, 71 | 0 | 0, 94 | 0 | 0, 125 | 0.14 |

^‡^Test for the difference using Kruskal Wallis test with correction for multiple comparisons, unlike superscript indicate differences (^*,†,§^)(overall p-value not displayed for milk and dairy products, red meat, white meat, processed meat products, lean, fatty fish and shellfish, fish products, and butter/margarine, no significant difference in post hoc test between consuming groups); Statistically significant values between the dietary groups < 0.05 are given in bold (two-sided); ^│^Not including processed/ prepared (fried) potatoes (included in the convenience food category); ^¶^ In addition to meat substitutes the food items ‘hummus’, ‘sesame paste, tahini’, ‘Vegetable pâté, Tartex’are included. For description of food items included in the food groups see **Supplemental Table 1**.
