# Supplementary material for: Food groups, macronutrient intake and objective measures of total carotenoids and fatty acids in 16-to-24-year-olds following different plant-based diets compared to an omnivorous diet
Source: PLoS One. 2025 Jan 17;20(1):e0311118. doi: 10.1371/journal.pone.0311118 (PMC11741618; doi:10.1371/journal.pone.0311118)
Supplement: S5 Table — (DOCX) [file pone.0311118.s005.docx]

| **Food groups, g/MJ** | **All**  **n = 165** | | **Vegans**  **n = 19** | | **Lacto-ovo-vegetarians**  **n = 20** | | **Pescatarians**  **n = 30** | | **Flexitarians**  **n = 25** | | **Omnivores**  **n = 71** | | **P-value** |
| --- | --- | --- | --- | --- | --- | --- | --- | --- | --- | --- | --- | --- | --- |
| **Plant-sourced foods** | **Median** | **25p,75p** | **Median** | **25p,75p** | **Median** | **25p,75p** | **Median** | **25p,75p** | **Median** | **25p,75p** | **Median** | **25p,75p** |  |
| Whole grain products, g/MJ^‡^ | 12 | 5, 18 | 13 | 6, 17 | 12 | 3, 19 | 11 | 8, 18 | 12 | 8, 21 | 9 | 2, 18 | 0.50 |
| Refined grain products, g/MJ^‡^ | 8 | 4, 14 | 10 | 5, 16 | 9 | 4, 12 | 11 | 4, 17 | 7 | 2, 12 | 7 | 4, 15 | 0.38 |
| Vegetables (all types), g/MJ^‡^ | 12 | 7, 20 | 22^*^ | 14, 29 | 13 | 8, 19 | 13 | 9, 20 | 14 | 10, 20 | 9^†^ | 3, 17 | **<0.001** |
| Fruit and berries (not including juice/smoothie), g/MJ^‡,a^ | 18 | 5, 33 | 22 | 9, 59 | 22 | 10, 36 | 12 | 0, 29 | 21 | 14, 34 | 14 | 0, 29 | **0.033** |
| Legumes, g/MJ^‡^ | 0 | 0, 4 | 5^*^ | 1, 15 | 4^*,†^ | 0, 7 | 0 | 0, 5 | 0^†,§^ | 0, 3 | 0^§^ | 0, 0 | **<0.001** |
| Nuts and seeds, g/MJ^‡^ | 0 | 0, 1 | 4^*^ | 0, 7 | 0^†^ | 0, 0 | 0^†^ | 0, 0 | 0^†^ | 0, 2 | 0^†^ | 0, 0 | **<0.001** |
| Vegetable oil, g/MJ^‡^ | 0 | 0, 0 | 0^*^ | 0, 1 | 0 | 0, 1 | 0 | 0, 1 | 0 | 0, 0 | 0^†^ | 0, 0 | **0.038** |
| Potatoes (including sweet potatoes), g/MJ^‡│^ | 0 | 0, 4 | 0 | 0, 3 | 0 | 0, 2 | 0 | 0, 6 | 2 | 0, 4 | 0 | 0, 5 | 0.58 |
| Vegetable products, g/MJ^‡^ | 1 | 0, 2 | 1 | 0, 7 | 1 | 0, 2 | 1 | 0, 3 | 0 | 0, 2 | 0 | 0, 1 | 0.18 |
| Fruit and berry products, g/MJ^‡^ | 0 | 0, 0 | 0^*^ | 0, 2 | 0 | 0, 0 | 0^†^ | 0, 0 | 0 | 0, 0 | 0^†^ | 0, 0 | **0.009** |
| Dairy product substitutes, g/MJ^‡^ | 0 | 0, 1 | 7^*^ | 1, 20 | 0^†,§^ | 0, 1 | 0^†,§^ | 0, 1 | 0^§^ | 0, 7 | 0^†^ | 0, 0 | **<0.001** |
| Meat substitutes and vegetarian food products, g/MJ^‡¶^ | 0 | 0, 3 | 7^*^ | 3, 14 | 3^*,†^ | 0, 4 | 0^†,§^ | 0, 5 | 0^†,§^ | 0, 1 | 0^§^ | 0, 0 | **<0.001** |
| Vegetarian dishes, g/MJ^‡^ | 0 | 0, 0 | 0 | 0, 0 | 0 | 0, 13 | 0 | 0, 0 | 0 | 0, 3 | 0 | 0, 0 | 0.14 |
| **Animal-sourced foods** |  |  |  |  |  |  |  |  |  |  |  |  |  |
| Milk and dairy products (including cheese), g/MJ^‡^ | 17 | 3, 34 | 0 | 0, 0 | 11 | 5, 35 | 17 | 3, 30 | 22 | 8, 34 | 24 | 8, 41 | **̶** |
| Eggs (all types), g/MJ^‡^ | 0 | 0, 6 | 0 | 0, 0 | 3 | 0, 9 | 0 | 0, 7 | 2 | 0, 8 | 1 | 0, 6 | **̶** |
| Red meat (all types), g/MJ^‡^ | 0 | 0, 3 | 0 | 0, 0 | 0 | 0, 0 | 0 | 0, 0 | 0 | 0, 5 | 3 | 0, 9 | **̶** |
| White meat (all types), g/MJ^‡^ | 0 | 0, 0 | 0 | 0, 0 | 0 | 0, 0 | 0 | 0, 0 | 0 | 0, 1 | 0 | 0, 5 | **̶** |
| Lean, fatty fish and shellfish, g/MJ^‡^ | 0 | 0, 7 | 0 | 0, 0 | 0 | 0, 0 | 0 | 0, 10 | 2 | 0, 10 | 0 | 0, 8 | **̶** |
| Fish products, g/MJ^‡^ | 0 | 0, 2 | 0 | 0, 0 | 0 | 0, 0 | 0 | 0, 5 | 0 | 0, 3 | 0 | 0, 4 | **̶** |
| Butter/margarine, g/MJ^‡^ | 0 | 0, 1 | 0 | 0, 0 | 0 | 0, 1 | 1 | 0, 1 | 0 | 0, 1 | 0 | 0, 1 | **̶** |
| **Sugary, salted and convenience foods** |  |  |  |  |  |  |  |  |  |  |  |  |  |
| Dessert, cake, and sweets, g/MJ^‡^ | 5 | 2, 11 | 2^*^ | 0, 5 | 6 | 3, 13 | 8^†^ | 3, 14 | 7 | 3, 12 | 4 | 1, 11 | **0.010** |
| Sweetened bread spread, g/MJ^‡^ | 0 | 0, 1 | 0 | 0, 1 | 0 | 0, 1 | 0 | 0, 1 | 0 | 0, 1 | 0 | 0, 1 | 0.75 |
| Sweetened cereal, g/MJ^‡^ | 0 | 0, 1 | 0 | 0, 0 | 0 | 0, 3 | 0 | 0, 0 | 0 | 0, 1 | 0 | 0, 2 | 0.38 |
| Salted snacks, g/MJ^‡^ | 0 | 0, 1 | 0 | 0, 2 | 0 | 0, 3 | 0 | 0, 2 | 0 | 0, 1 | 0 | 0, 1 | 0.75 |
| Convenience foods, g/MJ^‡^ | 0 | 0, 14 | 0^*^ | 0, 0 | 1 | 0, 14 | 0 | 0, 17 | 0 | 0, 11 | 7^†^ | 0, 20 | **0.021** |
| **Beverages** |  |  |  |  |  |  |  |  |  |  |  |  |  |
| Alcoholic beverages, g/MJ^‡^ | 0 | 0, 0 | 0 | 0, 0 | 0 | 0, 0 | 0 | 0, 0 | 0 | 0, 3 | 0 | 0, 0 | 0.73 |
| Non-sugary beverages, g/MJ^‡^ | 0 | 0, 24 | 0 | 0, 29 | 0 | 0, 6 | 3 | 0, 37 | 0 | 0, 0 | 0 | 0, 25 | 0.19 |
| Juice and smoothie, g/MJ^‡^ | 0 | 0, 10 | 5 | 0, 15 | 0 | 0, 17 | 0 | 0, 9 | 4 | 0, 10 | 0 | 0, 10 | 0.78 |
| Sugar-sweetened beverages, g/MJ^‡^ | 0 | 0, 12 | 0 | 0, 0 | 0 | 0, 15 | 0 | 0, 8 | 0 | 0, 13 | 0 | 0, 19 | 0.12 |

**Supplemental Table 5. Median energy-adjusted food group intake among Norwegian youth with different dietary practice**

^‡^Test for the difference using Kruskal Wallis test with correction for multiple comparisons, unlike superscript indicate differences (^*,†,§^); Statistically significant values between the dietary groups < 0.05 are given in bold (two-sided)**;** ^a^Non-significant in post-hoc test adjusted for multiple comparison; ^│^Not including processed/ prepared (fried) potatoes (included in the convenience food category); ^¶^ In addition to meat substitutes the food items ‘hummus’, ‘sesame paste, tahini’, ‘Vegetable pâté, Tartex’are included. For description of food items included in the food groups see **Supplemental Table 1.**
