# Supplementary material for: Food groups, macronutrient intake and objective measures of total carotenoids and fatty acids in 16-to-24-year-olds following different plant-based diets compared to an omnivorous diet
Source: PLoS One. 2025 Jan 17;20(1):e0311118. doi: 10.1371/journal.pone.0311118 (PMC11741618; doi:10.1371/journal.pone.0311118)
Supplement: S6 Table — (DOCX) [file pone.0311118.s006.docx]

**Supplemental Table 6. Mean absolute food group intake stratified by sex within dietary practice**

| **Food groups, g/d** | **All**  **Females**  **n = 125** | **All**  **Males**  **n = 40** | **P** | **Vegan**  **Females**  **n = 13** | **Vegan**  **Males**  **n = 6** | **Pescatarian**  **Females**  **n =26** | **Pescatarian**  **Males**  **n = 4** | **Flexitarian**  **Females**  **n = 22** | **Flexitarian**  **Males**  **n =3** | **Omnivores**  **Female**  **n= 45** | **Omnivores**  **Males**  **n = 26** |
| --- | --- | --- | --- | --- | --- | --- | --- | --- | --- | --- | --- |
| **Plant-sourced foods** | **Mean ± SD** | **Mean ± SD** |  | **Mean ± SD** | **Mean ± SD** | **Mean ± SD** | **Mean ± SD** | **Mean ± SD** | **Mean ± SD** | **Mean ± SD** | **Mean ± SD** |
| Whole grain products, g/d^‡^ | 90 ± 60 | 78 ± 91 | **0.039** | 76 ± 55 | 149 ± 129 | 102 ± 59 | 48 ± 37 | 96 ± 63 | 94 ± 65 | 89 ± 64 | 68 ± 85 |
| Refined grain products, g/d^‡^ | 63 ± 56 | 102 ± 89 | **0.010** | 67 ± 49 | 189 ± 128 | 82 ± 58 | 53 ± 34 | 51 ± 52 | 112 ± 86 | 60 ± 62 | 92 ± 75 |
| Vegetables (all types), g/d^‡^ | 104 ± 79 | 96 ± 90 | 0.29 | 181 ± 130 | 184 ± 93 | 115 ± 72 | 137 ± 90 | 96 ± 63 | 172 ± 106 | 86 ± 66 | 65 ± 69 |
| Fruit and berries, g/d^‡^ | 157 ± 131 | 131 ± 200 | **0.005** | 249 ± 226 | 306 ± 286 | 139 ± 117 | 0 ± 0 | 160 ± 110 | 234 ± 296 | 146 ± 121 | 104 ± 162 |
| Legumes, g/d^‡^ | 19 ± 31 | 20 ± 59 | **0.005** | 46 ± 43 | 84 ± 120 | 22 ± 33 | 41 ± 83 | 9 ± 16 | 38 ± 44 | 9 ± 21 | 0 ± 0 |
| Nuts and seeds, g/d^‡^ | 6 ± 12 | 14 ± 28 | 0.92 | 24 ± 23 | 58 ± 43 | 3 ± 7 | 14 ± 28 | 6 ± 12 | 8 ± 13 | 2 ± 6 | 5 ± 15 |
| Vegetable oil, g/d^‡^ | 2 ± 4 | 2 ± 4 | 0.26 | 6 ± 8 | 4 ± 4 | 2 ± 2 | 1 ± 3 | 1 ± 2 | 3 ± 6 | 1 ± 3 | 2 ± 4 |
| Potatoes and sweet potatoes, g/d^‡,│^ | 17 ± 29 | 26 ± 44 | 0.62 | 16 ± 29 | 14 ± 26 | 16 ± 25 | 51 ± 61 | 20 ± 32 | 27 ± 9 | 20 ± 33 | 26 ± 48 |
| Vegetable products, g/d^‡^ | 13 ± 25 | 19 ± 30 | 0.88 | 31 ± 57 | 36 ± 42 | 14 ± 18 | 29 ± 53 | 14 ± 30 | 13 ± 22 | 7 ± 12 | 15 ± 23 |
| Fruit and berry products, g/d^‡^ | 3 ± 15 | 5 ± 13 | 0.36 | 9 ± 23 | 19 ± 26 | 2 ± 6 | 0 ± 0 | 1 ± 3 | 1 ± 3 | 3 ± 14 | 2 ± 8 |
| Dairy product substitutes, g/d^‡^ | 30 ± 106 | 11 ± 38 | **0.017** | 147 ± 276 | 72 ± 76 | 14 ± 26 | 0 ± 0 | 43 ± 92 | 0 ± 0 | 4 ± 15 | 0 ± 0 |
| Meat substitutes, g/d^‡¶^ | 20 ± 40 | 17 ± 62 | **0.023** | 70 ± 68 | 101 ± 137 | 20 ± 32 | 15 ± 29 | 14 ± 38 | 0 ± 0 | 7 ± 23 | 0 ± 0 |
| Vegetarian dishes, g/d^‡^ | 35 ± 150 | 31 ± 190 | **0.005** | 30 ± 60 | 0 ± 0 | 67 ± 313 | 312 ± 592 | 14 ± 27 | 0 ± 0 | 22 ± 44 | 0 ± 0 |
| **Animal-sourced foods** |  |  |  |  |  |  |  |  |  |  |  |
| Milk and dairy products, g/d^‡^ | 152 ± 166 | 173 ± 216 | 0.99 | 0 ± 2 | 0 ± 0 | 141 ± 119 | 218 ± 234 | 142 ± 100 | 156 ± 147 | 218 ± 210 | 213 ± 234 |
| Eggs (all types), g/d^‡^ | 25 ± 36 | 25 ± 50 | 0.13 | 2 ± 9 | 0 ± 0 | 29 ± 46 | 6 ± 12 | 28 ± 41 | 41 ± 66 | 25 ± 30 | 33 ± 57 |
| Red meat (all types), g/d^‡^ | 13 ± 28 | 44 ± 68 | **0.005** | 0 ± 1 | 0 ± 0 | 0 ± 0 | 2 ± 4 | 19 ± 30 | 3 ± 5 | 27 ± 37 | 67 ± 74 |
| White meat (all types), g/d^‡^ | 6 ± 15 | 22 ± 53 | 0.12 | 0 ± 0 | 0 ± 0 | 0 ± 0 | 6 ± 13 | 8 ± 16 | 35 ± 61 | 16 ± 21 | 33 ± 63 |
| Lean, fatty fish and shellfish, g/d^‡^ | 30 ± 53 | 25 ± 47 | 0.30 | 0 ± 0 | 0 ± 0 | 44 ± 65 | 61 ± 93 | 42 ± 57 | 65 ± 57 | 37 ± 55 | 21 ± 40 |
| Fish products, g/d^‡^ | 20 ± 46 | 13 ± 33 | 0.13 | 0 ± 0 | 0 ± 0 | 30 ± 63 | 27 ± 42 | 28 ± 46 | 0 ± 0 | 25 ± 47 | 16 ± 37 |
| Butter/margarine, g/d^‡^ | 5 ± 12 | 5 ± 8 | 0.40 | 3 ± 7 | 1 ± 2 | 5 ± 7 | 13 ± 10 | 8 ± 27 | 2 ± 4 | 4 ± 4 | 4 ± 9 |
| **Sugary, salted and convenience foods** |  |  |  |  |  |  |  |  |  |  |  |
| Dessert, cake, and sweets, g/d^‡^ | 46 ± 47 | 84 ± 106 | 0.08 | 25 ± 32 | 20 ± 31 | 63 ± 54 | 92 ± 49 | 45 ± 37 | 65 ± 20 | 42 ± 49 | 98 ± 126 |
| Sweetened bread spread, g/d^‡^ | 4 ± 9 | 6 ± 12 | 0.94 | 6 ± 14 | 6 ± 11 | 5 ± 10 | 7 ± 10 | 4 ± 8 | 10 ± 13 | 3 ± 8 | 6 ± 14 |
| Sweetened cereal, g/d^‡^ | 7 ± 15 | 8 ± 22 | 0.34 | 6 ± 15 | 8 ± 21 | 3 ± 10 | 10 ± 20 | 9 ± 20 | 4 ± 7 | 10 ± 17 | 9 ± 25 |
| Salted snacks, g/d^‡^ | 7 ± 15 | 15 ± 30 | 0.92 | 5 ± 8 | 34 ± 41 | 7 ± 11 | 9 ± 12 | 8 ± 22 | 18 ± 31 | 8 ± 17 | 11 ± 29 |
| Convenience foods, g/d | 55 ± 97 | 135 ± 179 | **0.012** | 9 ± 21 | 17 ± 41 | 81 ± 131 | 129 ± 186 | 36 ± 64 | 25 ± 43 | 65 ± 97 | 158 ± 177 |
| **Beverages** |  |  |  |  |  |  |  |  |  |  |  |
| Alcoholic beverages, g/d^‡^ | 33 ± 101 | 41 ± 136 | 0.68 | 42 ± 150 | 41 ± 101 | 22 ± 79 | 188 ± 375 | 63 ± 163 | 0 ± 0 | 26 ± 63 | 25 ± 77 |
| Non-sugary beverages, g/d^‡^ | 120 ± 255 | 160 ± 315 | 0.83 | 70 ± 139 | 194 ± 268 | 140 ± 188 | 0 ± 0 | 88 ± 221 | 0 ± 0 | 155 ± 342 | 201 ± 360 |
| Juice and smoothie, g/d^‡^ | 50 ± 82 | 61 ± 135 | 0.84 | 52 ± 90 | 85 ± 83 | 43 ± 82 | 78 ± 124 | 39 ± 52 | 42 ± 38 | 54 ± 87 | 58 ± 157 |
| Sugar-sweetened beverages, g/d^‡^ | 50 ± 94 | 151 ± 254 | 0.13 | 7 ± 17 | 30 ± 75 | 45 ± 94 | 104 ± 208 | 42 ± 61 | 59 ± 52 | 71 ± 118 | 184 ± 290 |

^‡^Test for the difference using Mann-Whitney U test as the data are not normally distributed, data are presented as mean ± standard deviation for comparison with available data (no descriptive statistics shown for lacto-ovo-vegetarian due to only one male participant). Data are shown for descriptive purpose, due to few participants when stratified by sex within the dietary practices, p-value are not shown within the dietary practices; Statistically significant values between the groups < 0.05 are given in bold (two-sided)**;** ^│^Not including processed/ prepared (fried) potatoes (included in the convenience food category); ^¶^ In addition to meat substitutes the food items ‘hummus’, ‘sesame paste, tahini’, ‘Vegetable pâté, Tartex’ are included. For description of food items included in the food groups see **Supplemental Table 1.**
