# Supplementary material for: Food groups, macronutrient intake and objective measures of total carotenoids and fatty acids in 16-to-24-year-olds following different plant-based diets compared to an omnivorous diet
Source: PLoS One. 2025 Jan 17;20(1):e0311118. doi: 10.1371/journal.pone.0311118 (PMC11741618; doi:10.1371/journal.pone.0311118)
Supplement: S7 Table — (DOCX) [file pone.0311118.s007.docx]

**Supplemental Table 7. Mean energy-adjusted food group intake stratified by sex within dietary practice**

| **Food groups, g/MJ** | **All**  **Females**  **n = 125** | **All**  **Males**  **n = 40** | **P** | **Vegan**  **Females**  **n = 13** | **Vegan**  **Males**  **n = 6** | **Pescatarian**  **Females**  **n =26** | **Pescatarian**  **Males**  **n = 4** | **Flexitarian**  **Females**  **n = 22** | **Flexitarian**  **Males**  **n =3** | **Omnivores**  **Female**  **n= 45** | **Omnivores**  **Males**  **n = 26** |
| --- | --- | --- | --- | --- | --- | --- | --- | --- | --- | --- | --- |
| **Plant-sourced foods** | **Mean ± SD** | **Mean ± SD** |  | **Mean ± SD** | **Mean ± SD** | **Mean ± SD** | **Mean ± SD** | **Mean ± SD** | **Mean ± SD** | **Mean ± SD** | **Mean ± SD** |
| Whole grain products, g/MJ^‡^ | 14 ± 9 | 8 ± 9 | **<0.001** | 12 ± 9 | 12 ± 7 | 14 ± 9 | 5 ± 4 | 15 ± 10 | 14 ± 15 | 13 ± 9 | 8 ± 10 |
| Refined grain products, g/MJ^‡^ | 9 ± 8 | 12 ± 12 | 0.28 | 10 ± 7 | 23 ± 23 | 11 ± 7 | 7 ± 5 | 7 ± 6 | 13 ± 9 | 9 ± 9 | 10 ± 8 |
| Vegetables (all types), g/MJ^‡^ | 15 ± 11 | 11 ± 11 | 0.76 | 26 ± 14 | 21 ± 12 | 15 ± 8 | 17 ± 11 | 15 ± 10 | 18 ± 7 | 13 ± 9 | 8 ± 9 |
| Fruit and berries, g/MJ^‡^ | 24 ± 20 | 12 ± 17 | **<0.001** | 37 ± 31 | 25 ± 19 | 19 ± 15 | 0 ± 0 | 24 ± 16 | 25 ± 24 | 23 ± 20 | 10 ± 15 |
| Legumes, g/MJ^‡^ | 3 ± 5 | 2 ± 6 | **0.003** | 7 ± 7 | 9 ± 12 | 3 ± 4 | 4 ± 8 | 1 ± 2 | 7 ± 9 | 1 ± 3 | 0 ± 0 |
| Nuts and seeds, g/MJ^‡^ | 1 ± 2 | 1 ± 3 | 0.99 | 3 ± 3 | 5 ± 3 | 0 ± 1 | 1 ± 3 | 1 ± 1 | 1 ± 3 | 0 ± 1 | 1 ± 2 |
| Vegetable oil, g/MJ^‡^ | 0 ± 1 | 0 ± 1 | 0.19 | 1 ± 1 | 0 ± 1 | 0 ± 0 | 0 ± 0 | 0 ± 0 | 0 ± 1 | 0 ± 0 | 0 ± 1 |
| Potatoes and sweet potatoes, g/MJ^‡│^ | 3 ± 5 | 3 ± 5 | 0.94 | 2 ± 4 | 2 ± 4 | 2 ± 3 | 6 ± 7 | 3 ± 5 | 3 ± 0 | 3 ± 7 | 3 ± 6 |
| Vegetable products, g/MJ^‡^ | 2 ± 4 | 2 ± 3 | 0.76 | 4 ± 8 | 4 ± 5 | 2 ± 2 | 3 ± 5 | 2 ± 6 | 1 ± 2 | 1 ± 2 | 2 ± 3 |
| Fruit and berry products, g/MJ^‡^ | 1 ± 4 | 1 ± 2 | 0.37 | 1 ± 3 | 3 ± 4 | 0 ± 1 | 0 ± 0 | 0 ± 1 | 0 ± 1 | 0 ± 2 | 0 ± 1 |
| Dairy product substitutes, g/MJ^‡^ | 4 ± 15 | 1 ± 3 | **0.013** | 23 ± 39 | 6 ± 6 | 2 ± 4 | 0 ± 0 | 5 ± 11 | 0 ± 0 | 1 ± 2 | 0 ± 0 |
| Meat substitutes, g/MJ^‡¶^ | 3 ± 6 | 1 ± 4 | **0.017** | 10 ± 10 | 8 ± 6 | 3 ± 4 | 1 ± 3 | 1 ± 3 | 0 ± 0 | 1 ± 4 | 0 ± 0 |
| Vegetarian dishes, g/MJ^‡^ | 5 ± 19 | 3 ± 19 | **0.005** | 5 ± 10 | 0 ± 0 | 9 ± 39 | 31 ± 58 | 3 ± 5 | 0 ± 0 | 3 ± 7 | 0 ± 0 |
| **Animal-sourced foods** |  |  |  |  |  |  |  |  |  |  |  |
| Milk and dairy products, g/MJ^‡^ | 23 ± 25 | 19 ± 21 | 0.30 | 0 ± 0 | 0 ± 0 | 19 ± 17 | 26 ± 31 | 23 ± 16 | 16 ± 16 | 32 ± 26 | 23 ± 21 |
| Eggs (all types), g/MJ^‡^ | 4 ± 6 | 3 ± 6 | 0.07 | 0 ± 1 | 0 ± 0 | 4 ± 6 | 1 ± 2 | 5 ± 7 | 4 ± 6 | 4 ± 5 | 4 ± 7 |
| Red meat (all types), g/MJ | 2 ± 4 | 5 ± 6 | **0.012** | 0 ± 0 | 0 ± 0 | 0 ± 0 | 0 ± 1 | 3 ± 5 | 0 ± 1 | 4 ± 5 | 7 ± 7 |
| White meat (all types), g/MJ^‡^ | 1 ± 3 | 3 ± 7 | 0.20 | 0 ± 0 | 0 ± 0 | 0 ± 0 | 1 ± 2 | 1 ± 2 | 0 ± 0 | 2 ± 4 | 4 ± 9 |
| Lean, fatty fish and shellfish, g/MJ^‡^ | 4 ± 7 | 2 ± 4 | 0.16 | 0 ± 0 | 0 ± 0 | 6 ± 7 | 6 ± 8 | 6 ± 9 | 6 ± 6 | 6 ± 8 | 2 ± 4 |
| Fish products, g/MJ^‡^ | 3 ± 6 | 1 ± 3 | 0.09 | 0 ± 0 | 0 ± 0 | 4 ± 7 | 3 ± 4 | 4 ± 7 | 0 ± 0 | 4 ± 7 | 1 ± 3 |
| Butter/margarine g/MJ^‡^ | 1 ± 1 | 1 ± 1 | 0.14 | 0 ± 1 | 0 ± 0 | 1 ± 1 | 2 ± 1 | 1 ± 1 | 0 ± 0 | 1 ± 1 | 1 ± 1 |
| **Sugary, salted and convenience foods** |  |  |  |  |  |  |  |  |  |  |  |
| Dessert, cake, and sweets, g/MJ^‡^ | 7 ± 6 | 9 ± 10 | 0.42 | 3 ± 4 | 3 ± 5 | 8 ± 6 | 12 ± 8 | 7 ± 5 | 8 ± 1 | 6 ± 7 | 9 ± 11 |
| Sweetened bread spread, g/MJ^‡^ | 1 ± 1 | 1 ± 2 | 0.91 | 1 ± 2 | 0 ± 1 | 1 ± 1 | 1 ± 1 | 1 ± 1 | 1 ± 1 | 1 ± 2 | 1 ± 2 |
| Sweetened cereal, g/MJ^‡^ | 7 ± 15 | 8 ± 22 | 0.32 | 1 ± 3 | 1 ± 2 | 0 ± 1 | 1 ± 3 | 1 ± 3 | 0 ± 1 | 1 ± 2 | 1 ± 2 |
| Salted snacks, g/MJ^‡^ | 1 ± 2 | 1 ± 3 | 0.92 | 1 ± 1 | 3 ± 3 | 1 ± 1 | 1 ± 2 | 1 ± 3 | 2 ± 3 | 1 ± 3 | 1 ± 3 |
| Convenience foods, g/MJ | 8 ± 15 | 17 ± 25 | **0.035** | 1 ± 4 | 2 ± 4 | 12 ± 19 | 13 ± 16 | 7 ± 13 | 5 ± 8 | 10 ± 14 | 19 ± 24 |
| **Beverages** |  |  |  |  |  |  |  |  |  |  |  |
| Alcoholic beverages, g/MJ^‡^ | 4 ± 12 | 5 ± 15 | 0.67 | 5 ± 19 | 6 ± 14 | 3 ± 9 | 16 ± 32 | 8 ± 19 | 0 ± 0 | 4 ± 8 | 4 ± 13 |
| Non-sugary beverages, g/MJ^‡^ | 19 ± 42 | 18 ± 37 | 0.60 | 11 ± 20 | 28 ± 42 | 20 ± 27 | 0 ± 0 | 14 ± 33 | 0 ± 0 | 25 ± 59 | 22 ± 41 |
| Juice and smoothie, g/MJ^‡^ | 7 ± 12 | 7 ± 13 | 0.59 | 8 ± 13 | 11 ± 12 | 6 ± 11 | 7 ± 12 | 6 ± 9 | 4 ± 3 | 8 ± 12 | 6 ± 14 |
| Sugar-sweetened beverages, g/MJ^‡^ | 7 ± 14 | 18 ± 30 | 0.18 | 1 ± 2 | 3 ± 7 | 6 ± 12 | 9 ± 18 | 6 ± 10 | 6 ± 5 | 10 ± 19 | 22 ± 34 |

^‡^Test for the difference using Mann-Whitney U test as the data are not normally distributed, data are presented as mean ± standard deviation for comparison with available data (no descriptive statistics shown for lacto-ovo-vegetarian due to only one male participant). Data are shown for descriptive purpose, due to few participants when stratified by sex within the dietary practices, p-value are not shown within the dietary practices; Statistically significant values between the groups < 0.05 are given in bold (two-sided)**;** ^│^Not including processed/ prepared (fried) potatoes (included in the convenience food category); ^¶^ In addition to meat substitutes the food items ‘hummus’, ‘sesame paste, tahini’, ‘Vegetable pâté, Tartex’ are included. For description of food items included in the food groups see **Supplemental Table 1.**
