# Supplementary material for: Food groups, macronutrient intake and objective measures of total carotenoids and fatty acids in 16-to-24-year-olds following different plant-based diets compared to an omnivorous diet
Source: PLoS One. 2025 Jan 17;20(1):e0311118. doi: 10.1371/journal.pone.0311118 (PMC11741618; doi:10.1371/journal.pone.0311118)
Supplement: S8 Table — (DOCX) [file pone.0311118.s008.docx]

**Supplemental Table 8. Mean intake of carotenoid-rich foods among Norwegian youth with different dietary practice.**

| **Carotenoid-rich food groups** | **All** | | **Vegans** | | **Lacto-ovo- vegetarians** | | **Pescatarians** | | **Flexitarians** | | **Omnivores** | | **P** |
| --- | --- | --- | --- | --- | --- | --- | --- | --- | --- | --- | --- | --- | --- |
|  | **Mean** | **SD** | **Mean** | **SD** | **Mean** | **SD** | **Mean** | **SD** | **Mean** | **SD** | **Mean** | **SD** |  |
| **Absolute intake, g/d** |  |  |  | |  | |  | |  | |  | |  |
| ß-carotene-rich foods^‡,§^ | 42 | 46 | 62 | 65 | 41 | 33 | 45 | 43 | 50 | 61 | 32 | 35 | 0.08 |
| α-carotene-rich foods^‡,§^ | 13 | 24 | 23 | 34 | 11 | 22 | 16 | 25 | 10 | 17 | 10 | 23 | 0.23 |
| ß-cryptoxanthin-rich foods^‡,§^ | 62 | 75 | 95 | 79 | 49 | 53 | 53 | 77 | 65 | 63 | 59 | 82 | 0.31 |
| Lycopene-rich foods^‡,§^ | 34 | 57 | 52 | 55 | 47 | 88 | 40 | 56 | 21 | 22 | 28 | 55 | 0.26 |
| Lutein+zeaxanthin-rich foods^‡,§^ | 14 | 23 | 21 | 20 | 11 | 12 | 9 | 15 | 20 | 35 | 12 | 24 | 0.28 |
| Total carotenoid-rich foods^‡,§^ | 163 | 131 | 253^*^ | 180 | 159 | 114 | 163 | 122 | 165 | 120 | 141^†^ | 121 | **0.025** |
| **Energy-adjusted, g/MJ** |  |  |  |  |  |  |  |  |  |  |  |  |  |
| ß-carotene-rich foods^‡,§^ | 6 | 6 | 8 | 7 | 7 | 6 | 6 | 6 | 6 | 5 | 5 | 5 | 0.13 |
| α-carotene-rich foods^‡,§^ | 2 | 4 | 3 | 4 | 3 | 6 | 3 | 6 | 1 | 2 | 2 | 4 | 0.44 |
| ß-cryptoxanthin-rich foods^‡,§^ | 9 | 11 | 12 | 11 | 8 | 8 | 6 | 8 | 9 | 9 | 8 | 12 | 0.42 |
| Lycopene-rich foods^‡,§^ | 5 | 8 | 6 | 7 | 8 | 14 | 5 | 7 | 3 | 4 | 4 | 8 | 0.29 |
| Lutein+zeaxanthin-rich foods^‡,§^ | 2 | 3 | 3 | 2 | 2 | 2 | 1 | 2 | 3 | 4 | 2 | 3 | 0.25 |
| Total carotenoid-rich foods^‡,§^ | 23 | 17 | 32 | 21 | 28 | 20 | 21 | 15 | 23 | 11 | 20 | 17 | 0.05 |

^‡^Test for the difference using one-way ANOVA with Bonferroni Post Hoc test with correction for multiple comparisons, unlike superscript indicate differences (^*,†^); Statistically significant values between the dietary groups <0.05 are given in bold (two-sided); ^§^ß-carotene-rich foods **=** carrot, broccoli, pepper, lettuce, leek, spinach, cantaloupe melon, chili (dried apricot and parsley not reported); α-carotene-rich foods = carrot; ß-cryptoxanthin-rich foods = orange juice, clementine, pepper, orange, corn, mango, watermelon, popcorn, chili, pineapple (peach and basil not reported); lycopene-rich foods = canned tomato, fresh tomato, ketchup, fresh cherry tomato, tomato puree, watermelon, boiled tomato, tomato soup powder; lutein+zeaxanthin-rich foods = broccoli, pepper, lettuce, leek, spinach, corn, corn meal, popcorn, peas, corn flour, brussels sprouts (basil, parsley, and cornflakes not reported); total carotenoid-rich foods = all carotenoid-rich food groups combined into a total carotenoid-rich food variable. Detailed descriptions of the food items included in the different food subcategories within the carotenoid-rich food groups are presented in **Supplemental Table 3.**
