# Supplementary material for: Food groups, macronutrient intake and objective measures of total carotenoids and fatty acids in 16-to-24-year-olds following different plant-based diets compared to an omnivorous diet
Source: PLoS One. 2025 Jan 17;20(1):e0311118. doi: 10.1371/journal.pone.0311118 (PMC11741618; doi:10.1371/journal.pone.0311118)
Supplement: S9 Table — (DOCX) [file pone.0311118.s009.docx]

| **Carotenoid-rich food groups** | **All** | | **Vegans** | | **Lacto-ovo-vegetarians** | | **Pescatarians** | | **Flexitarians** | | **Omnivores** | | **P-value** |
| --- | --- | --- | --- | --- | --- | --- | --- | --- | --- | --- | --- | --- | --- |
|  | **Median** | **25^th^,75^th^** | **Median** | **25^th^,75^th^** | **Median** | **25^th^,75^th^** | **Median** | **25^th^,75^th^** | **Median** | **25^th^,75^th^** | **Median** | **25^th^,75^th^** |  |
| **Absolute intake, g/d** |  |  |  | |  | |  | |  | |  | |  |
| ß-carotene-rich foods^‡,§^ | 28 | 10, 55 | 37 | 13, 74 | 32 | 18, 50 | 36 | 13, 61 | 35 | 13, 49 | 21 | 3, 55 | 0.09 |
| α-carotene-rich foods^‡,§^ | 0 | 0, 15 | 5 | 0, 51 | 2 | 0, 10 | 4 | 0, 23 | 0 | 0, 16 | 0 | 0, 14 | 0.14 |
| ß-cryptoxanthin-rich foods^‡,§,a^ | 27 | 0, 103 | 88 | 30, 174 | 35 | 6, 59 | 12 | 0, 90 | 43 | 13, 130 | 17 | 0, 100 | **0.042** |
| Lycopene-rich foods^‡,§^ | 14 | 0, 46 | 32 | 4, 98 | 29 | 0, 42 | 23 | 0, 56 | 13 | 1, 41 | 5 | 0, 38 | 0.15 |
| Lutein+zeaxanthin-rich foods^‡,§^ | 4 | 0, 19 | 12^*^ | 4, 37 | 7 | 0, 20 | 0 | 0, 16 | 5 | 0, 19 | 0^†^ | 0, 16 | **0.031** |
| Total carotenoid-rich foods^‡,§^ | 136 | 61, 239 | 241 | 83, 353 | 122 | 94, 209 | 128 | 63, 231 | 138 | 65, 233 | 97 | 37, 230 | 0.06 |
| **Energy-adjusted, g/MJ** |  |  |  |  |  |  |  |  |  |  |  |  |  |
| ß-carotene-rich foods^‡,§,a^ | 4 | 2, 8 | 6 | 2, 13 | 6 | 3, 9 | 5 | 2, 8 | 5 | 2, 8 | 3 | 0, 7 | **0.033** |
| α-carotene-rich foods^‡,§^ | 0 | 0, 2 | 1 | 0, 6 | 0 | 0, 2 | 1 | 0, 2 | 0 | 0, 3 | 0 | 0, 2 | 0.17 |
| ß-cryptoxanthin-rich foods^‡,§,a^ | 5 | 0, 14 | 10 | 3, 18 | 6 | 1, 15 | 2 | 0, 10 | 6 | 2, 15 | 2 | 0, 15 | **0.037** |
| Lycopene-rich foods^‡,§^ | 2 | 0, 6 | 4 | 1, 10 | 4 | 0, 8 | 4 | 0, 7 | 2 | 0, 6 | 1 | 0, 5 | 0.16 |
| Lutein+zeaxanthin-rich foods^‡,§^ | 1 | 0, 3 | 3^*^ | 1, 4 | 1 | 0, 4 | 0 | 0, 2 | 1 | 0, 3 | 0^†^ | 0, 2 | **0.017** |
| Total carotenoid-rich foods^‡,§^ | 19 | 9, 33 | 25 | 18, 44 | 22 | 13, 37 | 17 | 9, 34 | 23 | 13, 29 | 17 | 5, 31 | 0.06 |

**Supplemental Table 9. Median intake of carotenoid-rich foods among Norwegian youth with different dietary practice.**

^‡^Test for the difference using Kruskal Wallis test with correction for multiple comparisons, unlike superscript indicate differences (^*,†^); Statistically significant values between the dietary groups <0.05 are given in bold (two-sided); ^a^Non-significant in post-hoc test adjusted for multiple comparison; ^§^*ß-carotene-rich foods* = carrot, broccoli, pepper, lettuce, leek, spinach, cantaloupe melon, chili (dried apricot and parsley not reported); *α-carotene-rich foods* = carrot; *ß-cryptoxanthin-rich foods* = orange juice, clementine, pepper, orange, corn, mango, watermelon, popcorn, chili, pineapple (peach and basil not reported); *lycopene-rich foods* = canned tomato, fresh tomato, ketchup, fresh cherry tomato, tomato puree, watermelon, boiled tomato, tomato soup powder; *lutein+zeaxanthin-rich foods* = broccoli, pepper, lettuce, leek, spinach, corn, corn meal, popcorn, peas, corn flour, brussels sprouts (basil, parsley, and cornflakes not reported); *total carotenoid-rich foods* = all carotenoid-rich food groups combined into a total carotenoid-rich food variable. Detailed description of food items included in the different food subcategories within the carotenoid-rich food groups are presented in **Supplemental Table 3**.
