# Supplementary material for: Food groups, macronutrient intake and objective measures of total carotenoids and fatty acids in 16-to-24-year-olds following different plant-based diets compared to an omnivorous diet
Source: PLoS One. 2025 Jan 17;20(1):e0311118. doi: 10.1371/journal.pone.0311118 (PMC11741618; doi:10.1371/journal.pone.0311118)
Supplement: Attachment — Submitted filename: Reviewer response.pdf [file pone.0311118.s010.pdf]

**Reviewer #1:**<br>Title: Food groups, macronutrient intake and objective measures of total carotenoids and fatty acids in 16-to-24-year-olds following different plant-based diets compared to an omnivorous diet<br><br>This study reports on the dietary intake of youths (aged 16-24 years) consuming different dietary patterns and compares the intake based on food groups, macronutrients, and blood sample analysis of carotenoids and fatty acids.<br><br>Overall, the manuscript is well-written and organized.<br><br>Some points to address:	Thank you for all the valuable remarks, we have tried our best to address all the comments point by point below.
Methods: Study Design and Study Population<br>• Can the authors provide a clear description of the timeline of the study? How much time elapsed between the food frequency questionnaire and the site visit with the first 24-hour dietary recall?<br>• Were blood samples collected at the same visit as the initial 24-hour dietary recall?	Thank you for this comment. On the first site visit, participants filled out the study consent, provided blood and urine samples, underwent body composition measurements, then completed the electronic questionnaire and the dietary screener. Subsequently, also at the first site visit, the initial 24-hour dietary recall was performed (interview administered).<br><br>Subsequently to the first site visit, participants received three self-administered 24-hour dietary recalls. The self-administered 24-hour dietary recalls were administered over an 8-week period.<br><br>We agree that more information should be added in regard to the study timeline for more clarity. Please see added lines, 94-102.
Methods: Classification into dietary practice<br>• Did participants also self-report their own dietary practice? How did it differ from their actual consumption?	Thank you for this comment, we also assessed participants self-identified dietary practice in the past six months. However, due to our inclusion criteria of adherence to their respective diet for a minimum of six months, we used their reported inclusion of animal source foods in the diet the past six months, in the electronic questionnaire, for categorization into the different dietary practices.<br>Previously self-identified dietary practices have also been found to deviate from reported consumption of animal source foods.<br><br>Deviation from the self-identified dietary practice with the categorization based on the reported consumption of animal source food the previous six months is outlined below.<br>- two participants reported to self-identify with vegan dietary practice, in which one these two was categorized as pescatarian (based on reported inclusion of seafood in the electronic questionnaire the past six months) and one was categorized as lacto-ovo-vegetarian (based on reported consumption of dairy or egg in the electronic questionnaire the past six months).<br>- two self-identified lacto-ovo-vegetarians were categorized as one omnivore (based on

	reported consumption of meat in the electronic questionnaire the previous six months) and one as pescatarian (based on reported consumption of seafood in the electronic questionnaire the previous six months).<br>- two self-identified ovo-vegetarians were categorized as lacto-ovo-vegetarians (as lacto-vegetarians, ovo-vegetarians and lacto-ovo-vegetarians are combined in the manuscript).<br>- one self-identified ovo-vegetarian was categorized as an omnivore (based on reported consumption of meat the previous six months).<br>- one self-identified pescatarian was categorized as an omnivore (based on reported consumption of meat the previous six months).<br>- two self-identified flexitarians were categorized as omnivores (reported consumption of meat more than two servings per week).<br><br>Additionally, we have restructured the section for classification into the dietary practices and added more information for a clearer description, please see revised section, lines 104-126.<br><br>Please also see description in the strength and limitation section, 597-602, 620-625.
Methods:<br>Dietary Assessment<br>• Line 89: While the authors note that the dietary assessment has previously been described elsewhere, can they provide a brief description here?<br>• Were participants given instructions on how to use Myfood24 during the initial recall?<br>• Approximately how long does it take for an individual to go through the Myfood24?	Thank you for this comment. We have restructured the 24-hour recall section for more clarity, please see the revised '24-hour dietary recalls' section, lines 127-158. In addition, we added information about approximate time to complete a recall, see added line 140 and 157-158.
Statistics<br>• Title line for Statistics should be moved to the next line.<br>• Line 159: "polysaturated" should be polyunsaturated<br>• Please include the value definitions of weak, moderate, and strong relationships regarding correlation coefficients.	Thank you for the concrete comments, this has now been corrected.<br><br>The correlations have been omitted from the aim, and the manuscript, as requested by the second reviewer.
Results:<br>• Table 2: Can the authors add separation between food groups to improve the visuals and a readability of the table? For example, group the plant-based foods, animal products, sweets, beverages, and alcohol.<br>• The authors report sex-differences as a limitation in the discussion. Could the authors further stratify their results based on sex for each dietary pattern? At a minimum, a descriptive analysis of the food groups and comparison to recommendations based on sex for energy intake would	Thank you for valuable comments, please see revised Table 2 and 3 for improvement of the readability of the tables. The tables are restructured according to 'plant-sourced foods', 'animal-sourced foods', 'sugar, salted and convenience foods', 'beverages'. We have also revised Supplemental Table 4-7 accordingly. Please also see the revised results section lines 238- 309 according to the revised tables.<br><br>We agree that it would be interesting to show data stratified based on sex within the dietary groups. For descriptive purposes, we have added supplemental Table 6 and 7 for food groups

make the report more interesting.	stratified by gender at group level and stratified by gender within the dietary groups in absolute intake (g/d) and energy-adjusted (g/MJ). Due to the power issues, from fewer participants in the plant-based dietary groups, test for difference split by sex within the dietary groups are not presented, only descriptive data.<br><br>Please see added lines in the method section, lines 216-219. Please also see, added lines in the result section, lines 242-243. Additionally, see added lines in the discussion, 524-534, and also added lines in the limitation section highlighting the power issue as the reason for presenting food group intake split by gender descriptively in supplemental materials, added lines 611-612.
Discussion:<br>• While the common definition of "youth" is 15-24 years, the average age in the report is 21 years (lowest was 20 and highest was 22). Much of the comparisons made are to studies with age ranges no higher than 18 years. This reviewer lacks knowledge on the cultural background of Norway – is there a large difference in lifestyle that could impact access to foods for those 16-18 versus those 19 and older? For example, those at university and living away versus those at home with family where food may be dictated by parental figures? Could these factors explain differences seen in these results versus those to which they are compared?<br>o Are there studies with more similar age ranges to which the authors can compare?	Thank you for your thorough review and valuable feedback. As of today, there is very limited data available from a youth age group as most studies assessing dietary intake among young age groups eating plant-based have been conducted on those 18 years or younger. The age range of the participants in this study is 16-24 years. We do acknowledge that a definition of the term 'youth should be added', please see added lines, 223-225.<br><br>According to statistics in Norway, the median age for moving out of their parental household is 19 years. Thus, those between 16-18 years might differ from those between 18-24 years, as those in the age range 16-18 years might still have help from their parental guardians to ensure an adequate and healthy diet.<br><br>During our revision process, we identified an error in the syntax of our analysis of the food item codes. This error resulted in overestimated intake values for all food groups. We have corrected the syntax error, and subsequently, all relevant sections of the manuscript have been revised to reflect the accurate data. The updated results are now more consistent with findings from previous studies. We appreciate your comments very much, which improve the accuracy of our manuscript.<br><br>Please see revised data in Table 2 and 3, and Supplemental Table 4-9. Accordingly see revised abstract. Please also see revised results lines 238-309 and revised discussion lines 404-419, 442-462, 476-534, 554-561. We have also revised the conclusion, accordingly, please see revised conclusion, lines 27-29 and 631-644.
Supplemental Information<br>• Supplemental Table 2 : Ingredients is misspelled as "ingrediens"	This has been corrected accordingly.
**Reviewer #2**<br>This study has two primary objectives. The first objective is to investigate the impact of different diets on the food intake of Nordic youth and assess the extent to which these diets comply with dietary guidelines. The second objective is to validate self-reported fruit and vegetable consumption by comparing it with objective blood measurements of carotenoids. The	Thank you for your valuable feedback helping to improve the quality of the manuscript. We have addressed these issues below point-by-point.

strength of the study is its use of objective measurements of blood dietary markers. Also, the article is well-written and flows well. However, major issues include attempting to address two distinct research questions in a single paper, insufficient explanation and discussion regarding the studied population aged 16-24 years, and the methods used for classifying participants' diets.	
Major Issues:<br>1) Addressing participants' diet selection on their food consumption and macronutrient intake, while also attempting to validate a questionnaire for the objective assessment of fruit and vegetable intake, involves two distinct methodologies and topics that require substantial detail. Although this paper attempts to cover both, it lacks sufficient explanation in the introduction regarding the rationale for validating the self-report measure. Additionally, the methodology section does not clearly specify which carotenoids were examined or which food groups were expected to correlate with specific types of carotenoids. Furthermore, the results of the second research question are only presented in a supplementary table, adding to the confusion for readers trying to follow the discussion on these two separate research questions. Therefore, I strongly recommend that the authors remove the second research aim from this paper and consider expanding on it to submit as a separate publication.	Thank you for this important comment. We agree that the secondary aim might lead to misunderstanding, as the aim of this paper is not to validate the dietary assessment tool (Myfood24) or the dietary screener used to assess their dietary intake. Therefore, we chose to omit the secondary objective and the correlations from the results section, the discussion section, and correlations presented in supplemental material. Please also see revised aim in the abstract and introduction, lines 4-6 and 72-74.
Major Issues:<br>2) This study included participants aged 16-24 years, but the rationale for selecting this age group is not provided in the introduction or methodology. This age range encompasses both adolescents and early adults. Based on the mean age presented in the results, it appears that most participants were college students (early adults). However, the discussion section predominantly focuses on children and adolescents, which does not accurately reflect the sample of this study. Therefore, I recommend adding a detailed explanation in the introduction regarding the selection of the sample and improving the discussion by incorporating studies conducted on college students and late adolescents.	Thank you for this comment. As of today, there is very limited data available from a youth age group as most studies assessing dietary intake among young age groups eating plant-based have been conducted on those at 18 years or younger. The United Nation (UN) defines youth as those who are between the age of 15-24 years. In the VeggiSkills-Norway project, in which the data is from, the term 'youth' is applied to describe those in the age group of 16-24 years based on the definition by the UN. The minimum age of 16 years was chosen for practical considerations, to allow youth to participate without written approval from their parental guardians. Please see added lines in the method in the ethics section, lines 223-228. Please also see added lines in the introduction, lines 64-66, 69-72.<br><br>In the discussion we have referred to both the systematic review on children and adolescents and adults consuming plant-based and omnivorous diets, however, we have chosen to highlight the findings from youth as the studies with adults have a large age range.<br><br>Please see references 16-18 (children and adolescents), reference 46 (adults), and revised discussion section with comparison to the general population in Norway with both adolescents and adults, lines 498-534.
Major Issues:	Thank you for this comment. Participants self-identified dietary practice the past six months

3) The biggest concern I have regarding the findings is the method used for classifying participants' diets (Lines 75-79). Researchers determined the participants' diets based on their responses to the food frequency questionnaire. However, this classification was not confirmed with the participants (or if it was, it was not clearly stated in the paper). Consequently, six participants were later identified as not adhering to their diet, which may not be due to inconsistency in their diet but rather incorrect initial classification. The authors need to justify that their classification method accurately represents the participants' actual diets.	was assessed. However, due to our inclusion criteria of adherence to their respective diet for a minimum of six months, we used their reported consumption of animal source foods in the electronic questionnaire the past six months for categorization into the different diet practices.<br><br>Previously self-identified dietary practices have also been found to deviate from reported consumption of animal source foods (see lines 623-625).<br><br>Deviation from the self-identified dietary practice with the categorization based on the reported consumption of animal source food the previous six months is outlined below.<br><br>- two participants reported to self-identify with vegan dietary practice, in which one these two was categorized as pescatarian (based on reported inclusion of seafood in the electronic questionnaire the past six months) and one was categorized as lacto-ovo-vegetarian (based on reported consumption of dairy or egg in the electronic questionnaire the past six months).<br>- two self-identified lacto-ovo-vegetarians were categorized as one omnivore (based on reported consumption of meat in the electronic questionnaire the previous six months) and one as pescatarian (based on reported consumption of seafood in the electronic questionnaire the previous six months).<br>- two self-identified ovo-vegetarians were categorized as lacto-ovo-vegetarians (as lacto-vegetarians, ovo-vegetarians and lacto-ovo-vegetarians are combined in the manuscript).<br>- one self-identified ovo-vegetarian was categorized as an omnivore (based on reported consumption of meat the previous six months).<br>- one self-identified pescatarian was categorized as an omnivore (based on reported consumption of meat the previous six months).<br>- two self-identified flexitarians were categorized as omnivores (reported consumption of meat more than two servings per week).<br><br>Additionally, we have restructured the section for classification into the dietary practices and added more information for a clearer description, please see revised section, lines 104-126.<br><br>Please also see description in the strength and limitation section, 597-602.
Minor Issues:<br>4) In the abstract (Line 9), provide what blood markers collected. Example: provided dried blood samples (DBS) to analyze blood fatty acids and carotenoids.	Thank you for this comment, this has now been revised accordingly, please see added line 9.
Minor Issues:<br>5) Line 20-21: The authors did not examine the causality between	Thank you for this comment, we have omitted this from the conclusion. Please see revised conclusion, line 27-29 and 631-644.

nutrition education and dietary intake. Therefore, they cannot assert that education might benefit participants to improve their diet. Instead, they should concentrate on identifying which diet group demonstrated superior outcomes in terms of healthy food intake and macronutrient consumption compared to others.	
Minor Issues:<br>6) In lines 46-47, the authors mention that the association between diet and food intake has already been studied, and in the following paragraph, they highlight the innovation of the current study, particularly in its focus on a Nordic youth sample. However, the rationale for why the results might differ in a Nordic youth sample is missing.	Thank you for this, we added information of the importance of being sufficiently equipped with the necessary competencies to meet their dietary requirements without the help of their parental guardians. And that the novelty of this study is that this is the first study to provide detailed information on dietary quality in a Norwegian youth population following plant-based diets compared to a comparison groups of omnivores, please see added lines 64-66, 69-72.
Minor Issues:<br>7) Again, there is no background regarding the research aim provided in line 56-57. I recommend authors to remove this aim.	Thank you for this comment, we have removed the secondary aim as recommended.
Minor Issues:<br>8) In line 81-84: What happened to those participants? Did you remove them from the analysis? Or how did you address their inconsistent diet?	This study is part of a larger cross-sectional project called VeggiSkills-Norway, as outlined in line 77, thus, participants were classified into the different dietary practices based on the reported inclusion of animal source food in the diet the previous six months in the electronic questionnaire and this information was cross-checked with the reported consumption of food groups in the dietary screener (assessing consumption of 33 selected food groups the previous six months, 'MinMatMåned 1.1'). This categorization was used in analysis before evaluation of the dietary intake data reported in the 24-hour dietary recalls:<br><br>Groufh-Jacobsen S, Larsson C, Van Daele W, et al. Food literacy and diet quality in young vegans, lacto-ovo vegetarians, pescatarians, flexitarians and omnivores. *Public Health Nutrition*. 2023;26(12):3051-3061. doi:10.1017/S1368980023002124<br><br>For this study, a more detailed and comprehensive evaluation of the participants dietary intake was performed, in which all of the 24-hour dietary recalls were evaluated. After evaluation of the 24-hour dietary recalls, six participants were found to report small inconsistencies with their dietary practice. These participants were not re-classified as we wanted to have consistencies across the dietary groups in the papers published in the VeggiSkills-project. However, we have tried our best to be as transparent as possible to report the discrepancies. Sensitivity analysis was also performed.<br><br>We agree that this information was not clearly written, please see added lines concerning evaluation of the 24-hour dietary recalls, lines 114-126. Please also see restructured section 'classification into dietary practice' as it is revised for more clarity, lines 104-126.<br><br>Please also see lines 469-474 in the discussion stating that since few participants in our study

	reported inconsistency with their dietary practice, it is considered to not have a substantial impact on our study results. Please also see lines 620-6255, in which this is highlighted in the limitation section. Inconsistencies with dietary practices have previously also been reported, in which this is stated and cited in line 625 (references 56 and 57).
Minor Issues:<br>9) In line 90, the paper provided for further details is under review. I can not look at the details of the method utilized for dietary assessment. If the cited paper is not published, the authors should provide details in this paper.	Please see revised section for more details of the study timeline 94-102, and for details of the dietary assessment, lines 127-140, 150-158.<br><br>The study has now been published, please see added reference number 25.<br><br>Reference added:<br>Groufh-Jacobsen S, Larsson C, Margerison C *et al.* (2024) Micronutrient intake and status in young vegans, lacto-ovo-vegetarians, pescatarians, flexitarians, and omnivores. *Eur J Nutr*.
10) Line 97-99: This should be presented in the results, not in the methods.	Thank you for pointing this out, this is moved to line 261-262.
12) Line 111-112: How many weekdays and weekends have each participant completed?	Thank you for this comment, this was not analyzed in this paper. However, during the administration of the 24-hour recalls, the researcher sent out 24-hour recalls on different days to ensure that participants completed 24-hour recalls from different weekdays and from both weekdays and weekend days over the eight-week period.
13) Line 113-118: I have a general concern regarding the selection of food groups, particularly the inclusion of the meat food group. This inclusion is problematic since participants categorized as vegan, lacto-ovo vegetarians, and pescatarians should not consume any meats. Therefore, there is no hypothesis associated with this food group, as the definition of the independent variable requires the dependent variable to be null. The results presented in Table 2 support this observation, as meat intake is either 0 or very low for the aforementioned groups, while it is higher in the other groups. I recommend that the authors provide a justification for their selection of food groups and consider including only foods that can be consumed in plant-based diets and omnivore diets. This adjustment will not only clarify the hypothesis but also reduce the number of models run, thereby increasing the power of the study.	Thank you for this comment. Our study sample consists of participants who includes animal-sourced foods to varying degree. Thus, for this study to report food habits among this population group it would be important to also report animal-sourced foods not consumed by vegans. If these food groups are not included, we then lack information on what food groups that are consumed among the comparison group of omnivores and how the plant-based dietary groups replace plant-based foods in comparison to them. It is also relevant to include the animal source foods to be able to compare the dietary intake of the participating omnivores with the general population. Therefore, we wish to keep the animal-sources food groups in the tables. However, as previously stated, we have tried our best to be transparent regarding the discrepancy of the dietary reporting highlighted in the limitation section.<br><br>However, we have omitted the overall p-value between groups, to present the data for descriptive purpose, please see revised table 2 and 3. Please also see added lines, 215-219.
14) Line 131: What types of carotenoids were analyzed?	Please see added lines 181-183.
15) Line 138: What types of fatty acids were analyzed?	Please see added lines 189-191.
16) In the statistics section, there is no justification or description of the sample size selection. While I understand that this is a secondary study and the original study may not have been powered specifically for this question, it's important to provide some insight into the extent to which this study has power for the proposed analysis with the included sample size for the cross-sectional study. I recommend including a statement	Thank you for this comment, please see added lines 80-88.

addressing this aspect.	
17) Line 247: The results are presented for each carotenoid level, yet the introduction does not provide a rationale for why and which types of carotenoids are related to youth dietary intake. If these results are to be presented, the authors should introduce the types of carotenoids and their relationship with child dietary intake.	Thank you for this comment, we have omitted the secondary aim, thus this is mentioned in the discussion. The carotenoids in this study are used as objective biomarkers of fruit and vegetable intake. Please see lines, 547-561.
18) As previously mentioned, the discussion would benefit from incorporating findings from younger adults, given the sample demographics of the current study.	Thank you for this comment, as previously mentioned, available data in a youth population following plant-based diets is limited. However, we have included the most recent systematic review on dietary intake in children and adolescents and in adults following plant-based diets (reference 17 and 46). We have chosen to highlight the studies that have addressed a youth population in more detail.<br><br>Please see revised section with more details compared to Norwegian adolescents and adults in the general populations, 498-534.
19) Line 414: Please revised as Finnish adults	This has been corrected.
20) Line 489-491: This study cannot draw such conclusions as there was no educational nutrition intervention. Moving forward, the authors should focus on offering recommendations regarding which types of diets show promise for promoting healthy dietary intake or what strategies they recommend to youth in different diet groups to enhance their dietary habits.	Thank you for this comment, we have removed this line. Please also see revised conclusion in the abstract, line 27-29 and the conclusion in the main text, line 631-645.
